# Moisture content prediction of cigar leaves air-curing process based on stacking ensemble learning model

**DOI:** 10.3389/fpls.2025.1553110

**Published:** 2025-03-26

**Authors:** Zhuoran Xing, Yaqi Shi, Kai Zhang, Songshuang Ding, Xiangdong Shi

**Affiliations:** ^1^ College of Tobacco Science, National Tobacco Cultivation and Physiology and Biochemistry Research Center, Key Laboratory for Tobacco Cultivation of Tobacco Industry, Henan Agricultural University, Zhengzhou, China; ^2^ Anhui Fermented Food Engineering Research Center, School of Food and Biological Engineering, Hefei University of Technology, Hefei, Anhui, China

**Keywords:** air-curing process, moisture content, stacking ensemble learning, SHAP, cigar leaf

## Abstract

**Introduction:**

Accurately determining the moisture content of cigar leaves during the air-curing process is crucial for quality preservation. Traditional measurement techniques are often subjective and destructive, limiting their practical application.

**Methods:**

In this study, we propose a stacking ensemble learning model for non-destructive moisture prediction, leveraging image-based analysis of naturally suspended cigar leaves. In this study, front and rear surface images of cigar leaves were collected throughout the air-curing process. Color and texture features were extracted from these images, and a filtering method was applied to remove redundant variables. To ensure optimal model selection, the entropy weight method was employed to comprehensively evaluate candidate machine learning models, leading to the construction of a stacking ensemble model. Furthermore, we applied the SHAP method to quantify the contribution of each input feature to the prediction results.

**Results:**

The stacking ensemble model, comprising MLP, RF, and GBDT as base learners and LR as the meta-learner, achieved superior prediction accuracy (*R* ^2^_test_ =0.989) and outperforms than traditional machine learning models (*R* ^2^_test_ ranged from 0.961 to 0.982). SHAP analysis revealed that front surface features (45.5%) and leaf features (38.5%) were the most influential predictors, with airing period (*AP*), *a*_f_^*^, *G*_f_, and *ASM*_f_ identified as key predictors.

**Conclusion:**

This study provides a feasible and scalable solution for real-time and non-destructive monitoring of cigar leaf moisture content, offering effective technical support for similar agricultural and food drying applications.

## 1 Introduction

Air-curing is a key link in the production process of cigar leaves, as an important medium in this process, the dynamic change of moisture content directly affects the enzymatic reaction intensity of cigar leaves, resulting in the change of color and morphology (Yang et al., 2024). The harvested cigar leaves are hung in the drying room, and the temperature and humidity in the drying room are artificially adjusted to dissipate the moisture of the leaves, operators usually rely on intuition to estimate the moisture content of cigar leaves, this method has the defects of strong subjectivity and poor accuracy (Zhao et al., 2024a). Traditional moisture content measurement methods such as hot air drying, although with high accuracy, can cause damage to leaves (Nirmaan et al., 2020). However, some new measurement methods, such as spectral detection method, provide non-destructive detection of the moisture content of agricultural products during the drying process, they all rely on relatively expensive spectral equipment and are difficult to apply in actual production (Arevalo-Ramirez et al., 2020; Wei et al., 2021).

The image collected by the digital camera contains the color and texture information of the leaves (Li et al., 2022), with the rise of computer vision and machine learning algorithms, it is possible to predict the moisture content of the leaves by analyzing the morphological changes of the image representation. Yang et al. (2023b) used the color and texture features of cigar leaf images during the air-curing process to construct a moisture content prediction model for different stages. The prediction results show that the machine learning model can better predict the moisture content of cigar tobacco leaves at the wilting and yellowing stages, while the prediction performance of the model is poor when the tobacco leaves enter the fixation stage. (Xing et al., 2024). selected the key features of cigar leaf images during air-curing by constructing a random forest (RF) model, the results showed that for cigar leaves in a flat state, color features contributed more to the prediction results of moisture content. In addition, the above researches showed that when the tobacco leaves entered the fixation period, the change of apparent morphology was concentrated in the main vein, and the image information of the main vein could not be fully obtained by image acquisition after the cigar leaves were artificially flattened, resulting in poor prediction effect of the model on the moisture content of cigar leaves in the later stage of air-curing. To address this issue, in this study, by collecting the front and back images of cigar leaves under the natural suspension state during the air-curing process, a more comprehensive image information of tobacco leaves is obtained under the condition that it is closest to the actual production form.

For the extracted numerous image features, choosing a suitable prediction model has become another important issue. Single machine learning algorithms such as linear regression (LR), SVR, and artificial neural networks are effective at modelling nonlinear data by fitting feature distributions to specific functions within large data spaces (Lu et al., 2023). However, as the number of features increases, the nonlinear relationship between the image features of cigar leaves during the air-curing process and their moisture content becomes more complex, making it challenging for a single model to provide an accurate fit. Ensemble learning models, which aggregate predictions from multiple base models, are particularly effective at handling high-dimensional nonlinear data (Dong et al., 2020). The commonly used ensemble learning methods include parallelized average bagging (Salam and Islam, 2020), serialized weighted boosting (Tao et al., 2024) and multi-model combination stacking (Singh et al., 2020). Unlike homogeneous ensemble methods based on tree models such as bagging and boosting, heterogeneous ensemble methods synthesize predictions from diverse base learners, achieving higher accuracy and reducing the risk of overfitting. Unlike the simple nonlinear model, whose interpretation method can be directly derived from the model formula itself, the stacking ensemble learning model has achieved excellent performance with multisource data due to the heterogeneity of the base model (Al Shamsi and Abdallah, 2023). However, this diversity also results in a more complex structure and reduced interpretability. For an ensemble learning algorithm with many variable parameters, the objective is not to decipher the ‘black box’ logic but to present viable solutions for predicting specific sample outcomes (Li, 2022). Shapley Additive exPlanations (SHAP) is a method proposed by Lundberg and Lee (2017) for postinterpretation of machine learning models. This method is applied to enhance the interpretability of the decision-making process of the model by analyzing the average change in the prediction results in the presence or absence of each characteristic variable (Cakiroglu et al., 2024). This method has been proven to be effective in the fields of animal husbandry and agricultural product processing (Fang et al., 2024; Yan et al., 2024).

To solve the above issue, our study aims to non-destructively detect the moisture content in tobacco leaves by capturing images of their front and rear surfaces while naturally being suspended during the air-curing process on the basis of a stacking ensemble learning model. In addition, to overcome the shortcomings of the stacking ensemble learning model in terms of interpretability and explore the contribution of different characteristics to the prediction of moisture content, we introduce the SHAP method to identify key feature variables that significantly influence the decision-making process. This approach provides a theoretical foundation for the intelligent air-curing of cigar leaves. The results also provide a new perspective for the estimation of moisture content in drying processes such as agricultural products and food.

## 2 Methodology

**Figure 1:**
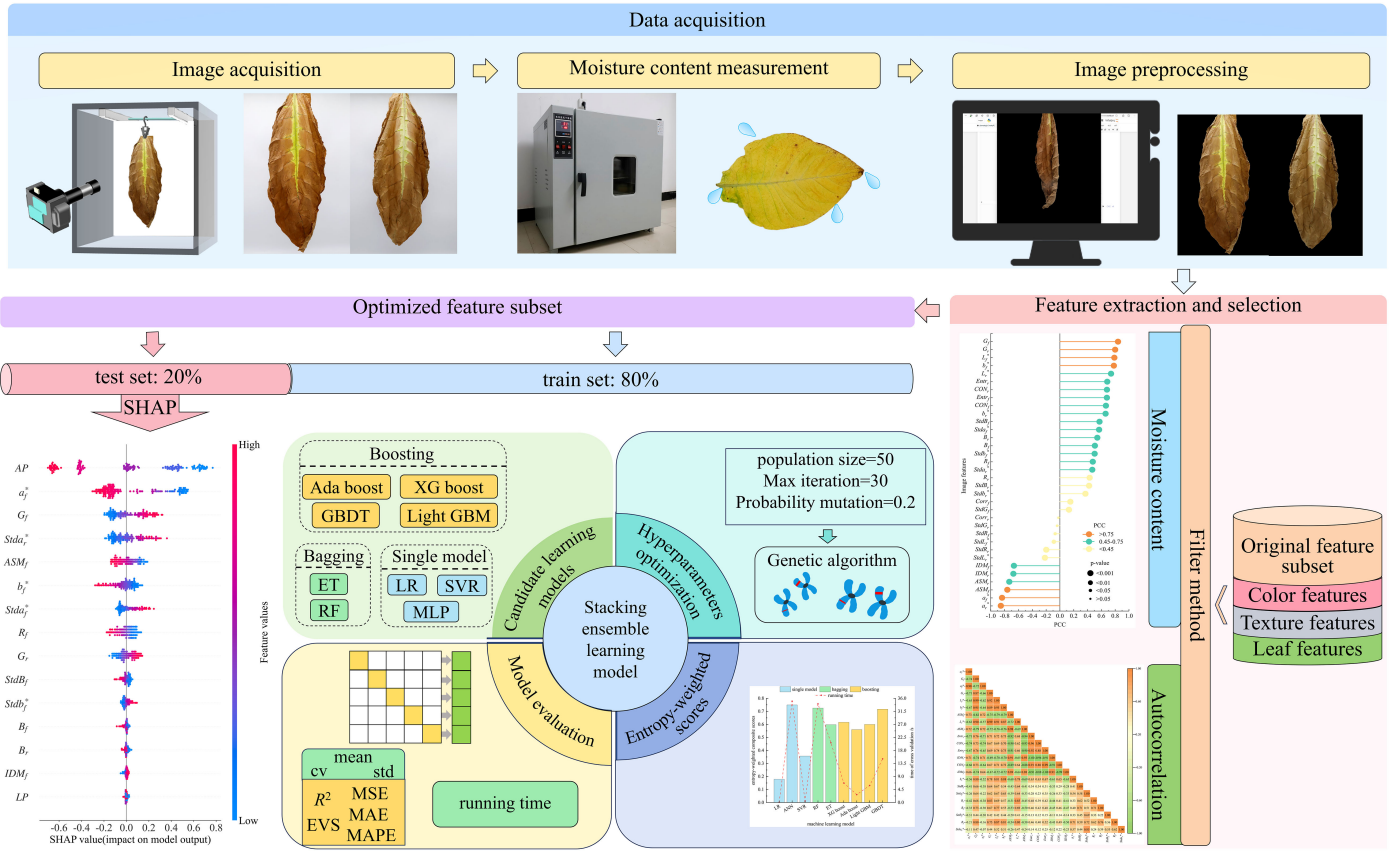
Flowchart of the cigar leaf moisture content prediction.

The workflow of this study is shown in **Figure 1**. First, a digital camera was used to capture images of the suspended cigar leaves. The region of interest (ROI) in the images were subsequently extracted to obtain color and texture features. In order to avoid the impact of collinearity on the model, the filtering method was used to select the optimized feature subset (Hou et al., 2023). Simultaneously, nine machine learning algorithms were employed as candidate base learners, comprising three single machine learning algorithms: LR, multi-layer perceptron (MLP), SVR, two Bagging algorithms: RF, extra tree (ET) regression, four boosting algorithms: gradient boosting decision tree (GBDT), extreme gradient boosting (XGBoost), adaptive boosting (AdaBoost), and light gradient boosting machine (LightGBM). The entropy weighting algorithm was used to compute the composite score from a 5-fold cross-validation of each candidate base learner, to construct the stacking ensemble learning model, which was then used to predict the test set. Finally, the SHAP method was applied to analyze how each input feature contributes to the model’s output.

### 2.1 Datasets

In this experiment, the images of cigar leaves under natural suspension during the whole air-curing process of the cigar variety ‘Yunxue-2’ were collected in Mayang, Huaihua City, Hunan Province. From the day of fresh cigar leaves harvest to the end of the air-curing process, the cigar leaves in the air-curing process were sampled every other day, and 20 cigar leaves in the same batch during the air-curing process were selected each time for image acquisition and moisture content determination. A total of 880 images of both the front and rear surfaces of cigar leaves image were collected. According to the difference of leaf position, the collected tobacco leaves included 280 lower leaves, 320 middle leaves, and 280 upper leaves. In addition, referring to the appearance factors such as the color and shape of the front and back of cigar leaves, and combining with the expert opinions on air-curing, the collected cigar leaves were divided into wilting period, yellowing period, browning period, fixation period and dry tendon period. Front and rear surface images of the cigar leaf during each air-curing period are displayed in **Table 1**. The image acquisition device is shown in **Supplementary Figure S1**, the size was 80 cm^3^ and features a white background board. Two 40 W white light tubes mounted on top of the dark box serve as the light source. A load-bearing rod with a clip attached to the vertical light tube was used to secure the leaf. The load-bearing rod is positioned 1.5 cm from the white background board to minimize light and shadow interference on the images. A digital camera (Canon company, Tokyo, Japan) was used for image acquisition. The resolution of all images was 2080×3120 and saved in the form of JPG.

**Table 1:** Cigar leaf shapes in the natural suspended state during different air-curing periods.

	Wilting period	Yellowing period	Browning period	Fixation period	Dry tendon period
Front surface of cigar leaf	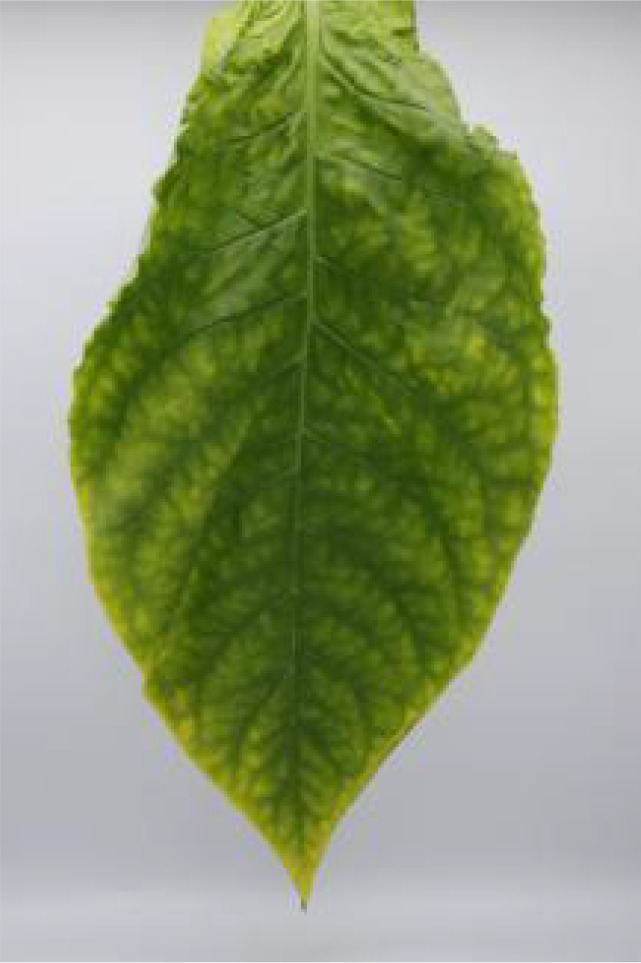	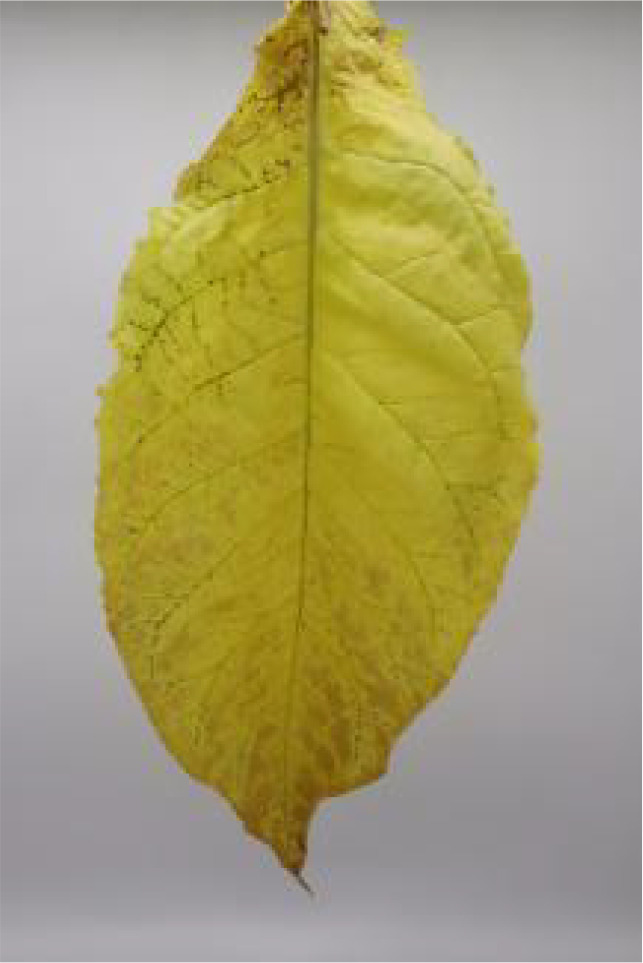	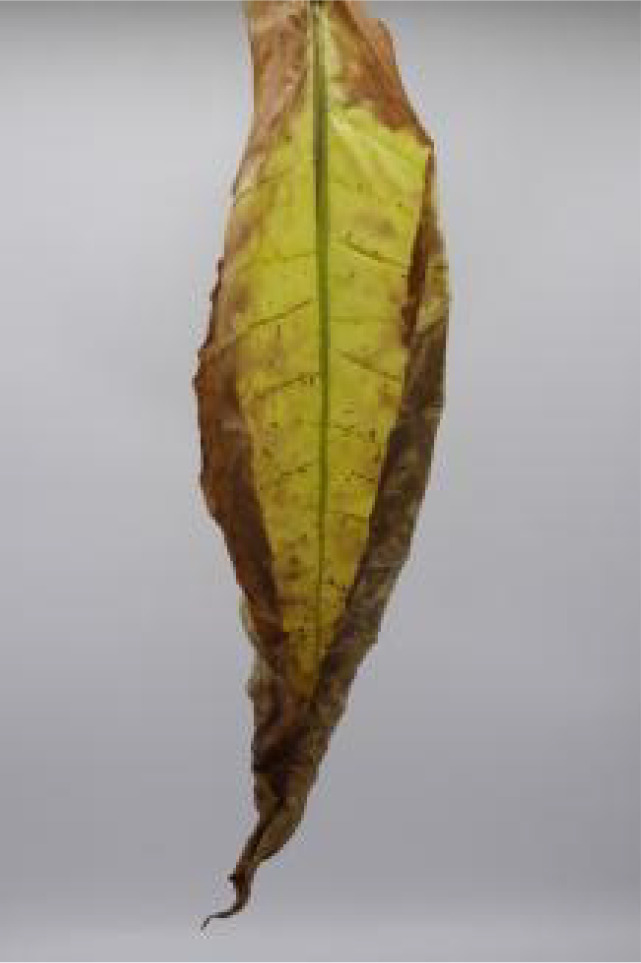	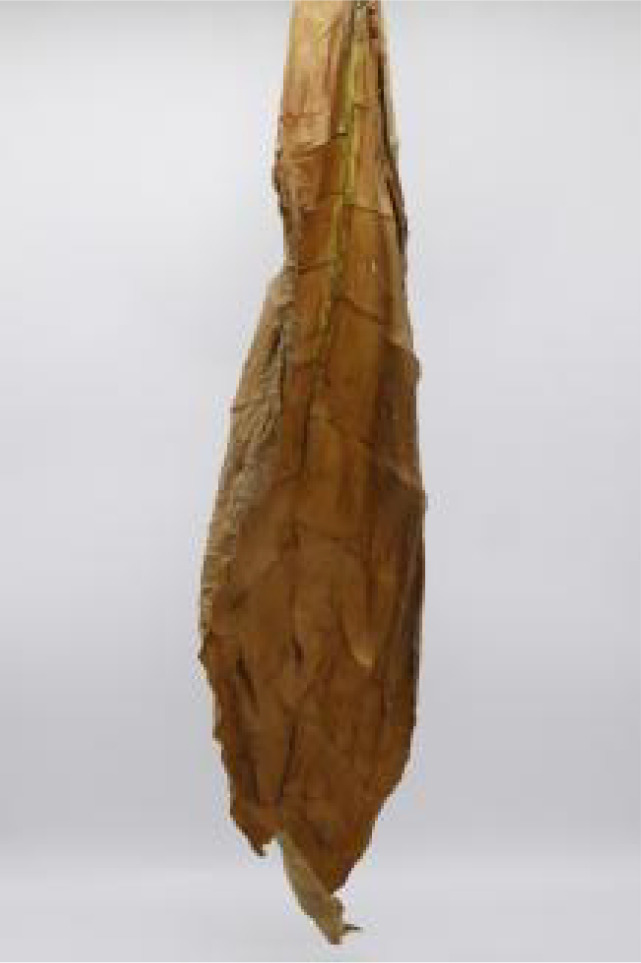	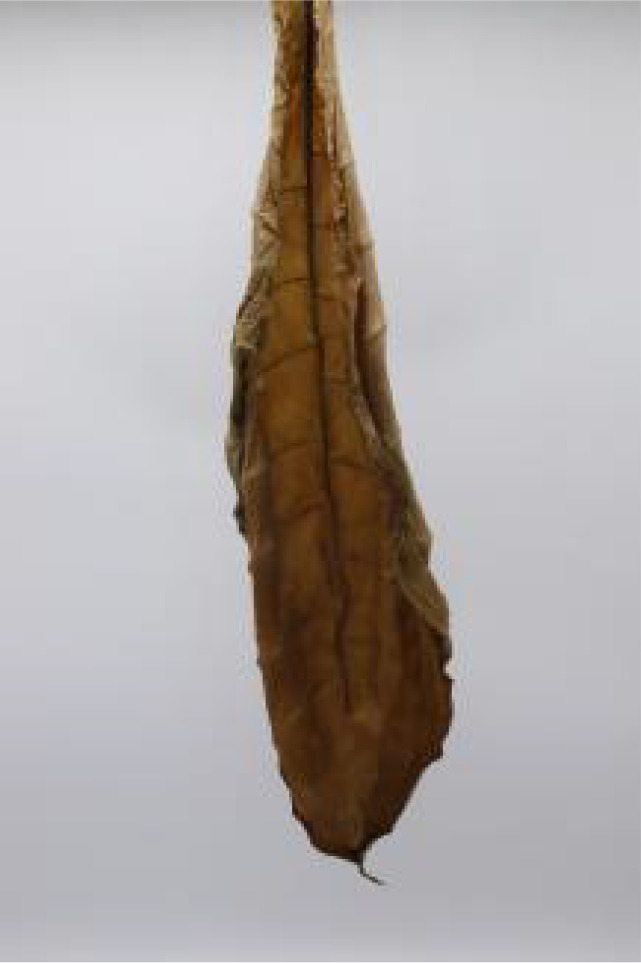
Rear surface of cigar leaf	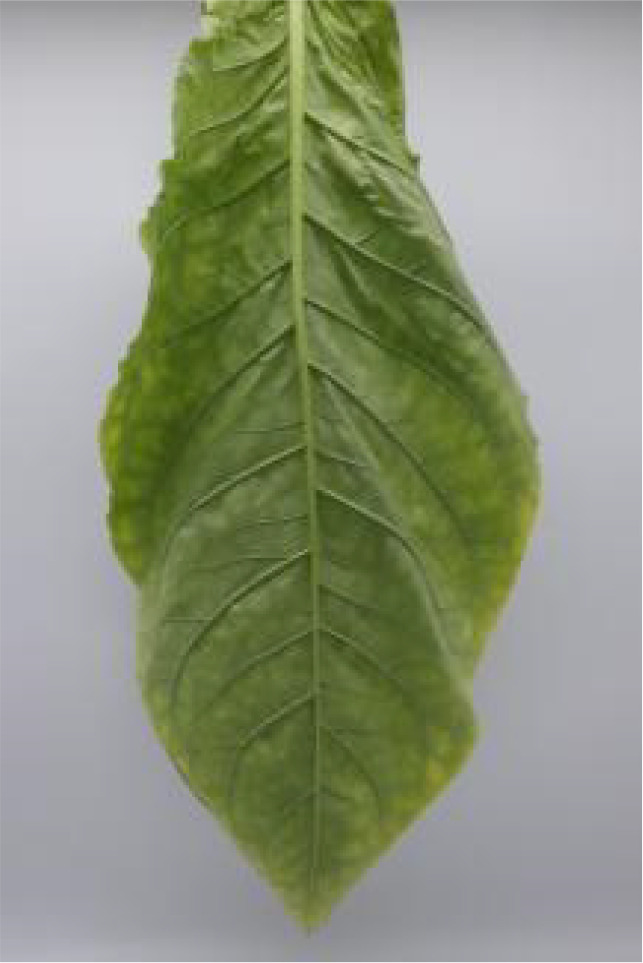	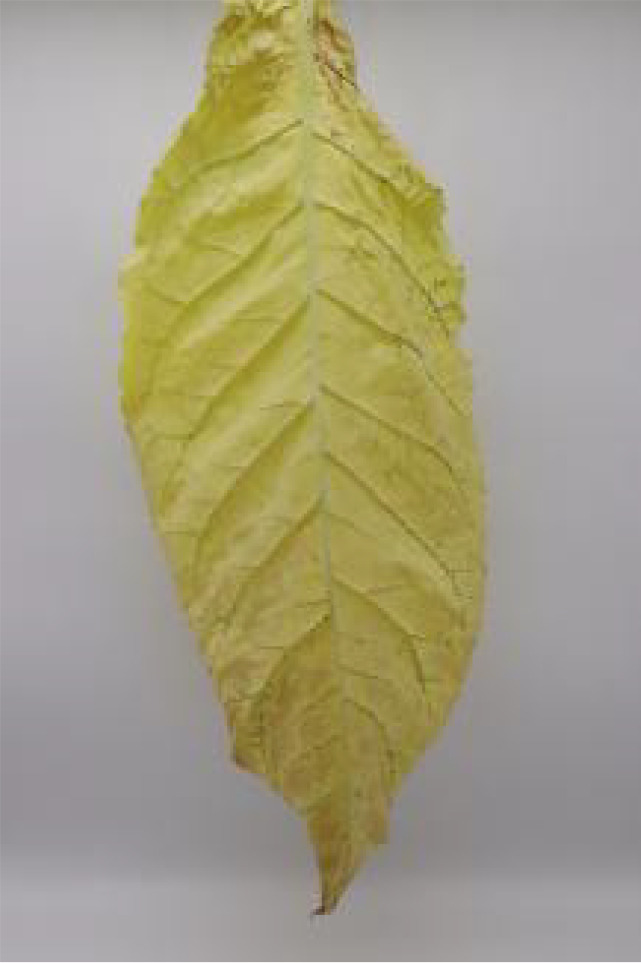	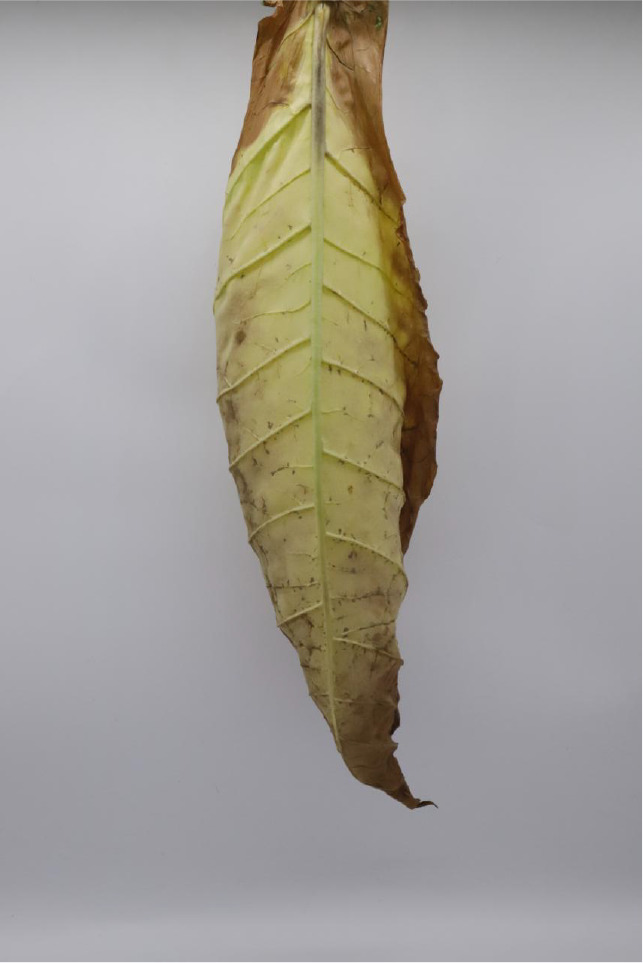	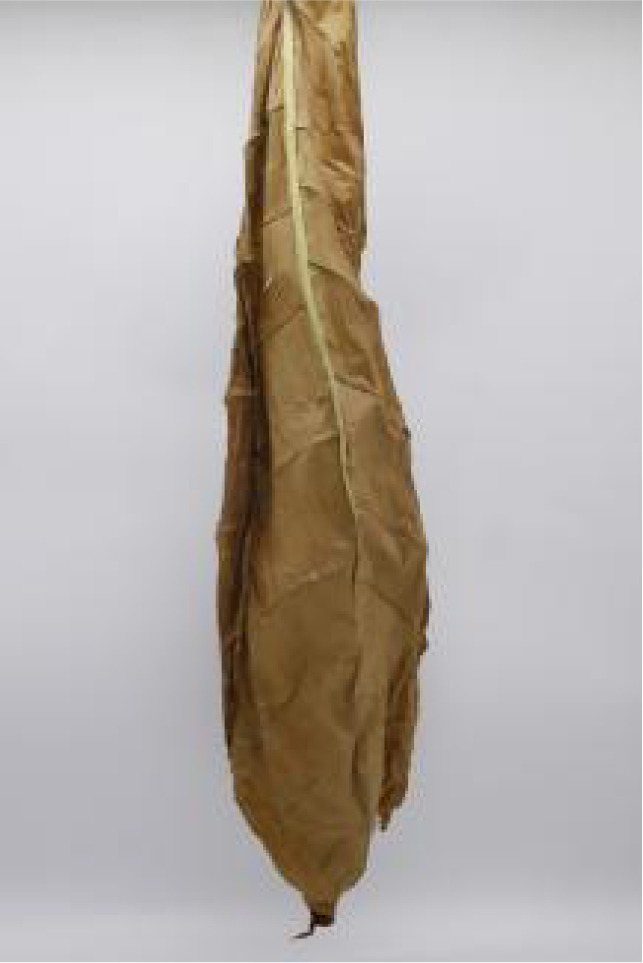	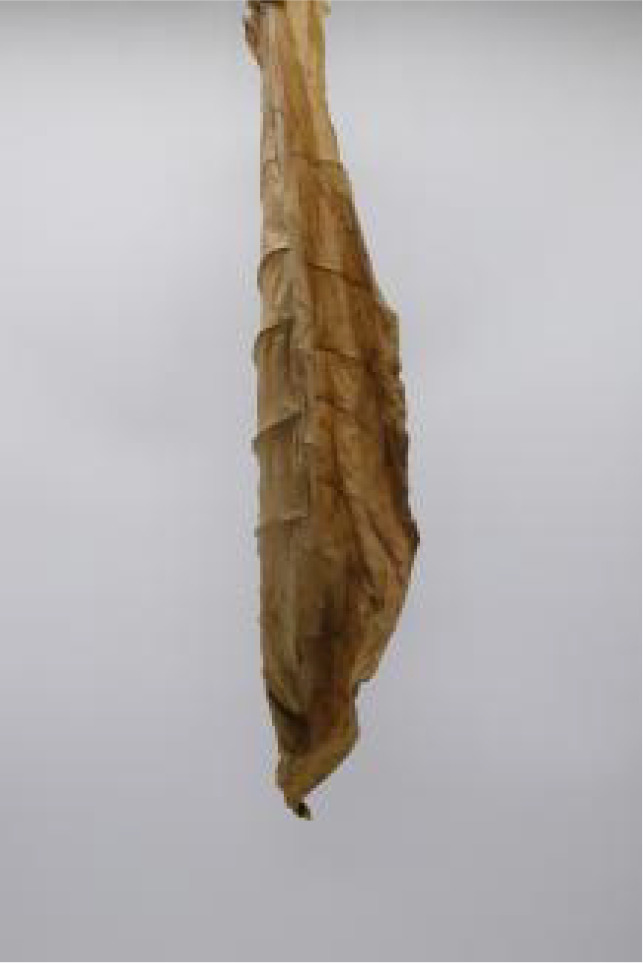

The method of measuring the moisture content of cigar leaves refers to the method of (Xu et al., 2018) The tobacco leaf is weighed once before the image acquisition, immediately after imaging, the tobacco leaves were placed in an oven and dried to constant weight, and the moisture content of tobacco leaves was calculated using Equation 1.


(1) $$\text{Moisture content }\left(\%\right)\;\frac{\left({\text{M}}_{\text{leaf}}{\text{-M}}_{0}\right)-\left({\text{M}}_{\text{dry}}{\text{-M}}_{0}\right)}{\left({\text{M}}_{\text{leaf}}{\text{-M}}_{0}\right)}$$

Where, *M_leaf_* represents the weight of tobacco leaves before drying, *M_dry_* represents the weight of tobacco leaves after drying, and *M*_0_ represents the weight of the container.

### 2.2 Image preprocessing and feature extraction

**Figure 2:**
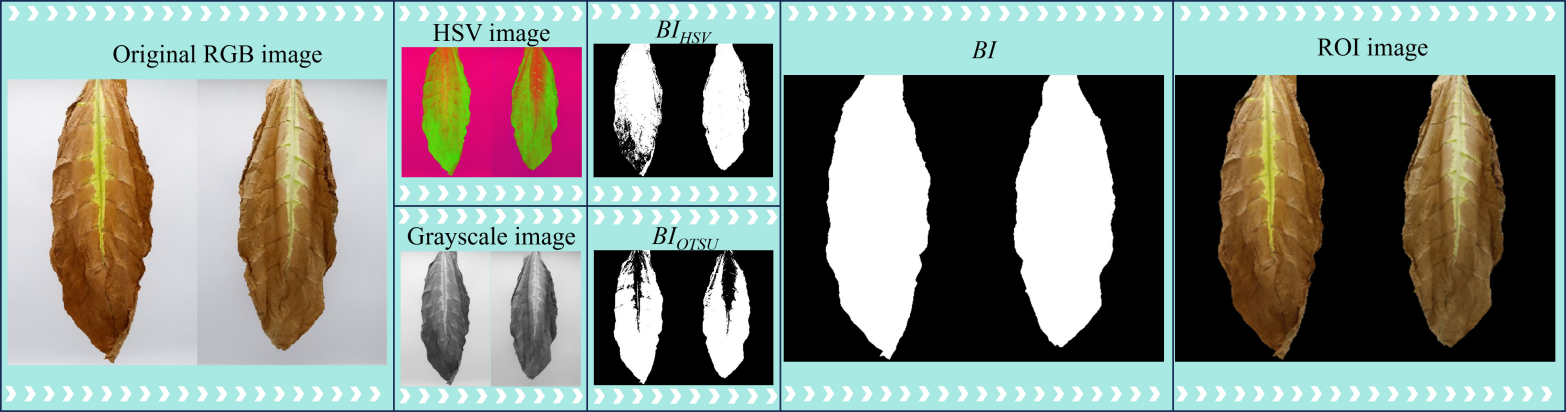
ROI extraction from cigar leaf images.


Before extracting features from the collected cigar leaf images, it is essential to preprocess the images to isolate ROI. Current mainstream image segmentation methods include deep learning-based approaches, clustering-based methods, grayscale thresholding segmentation, and color space-based segmentation. Deep learning methods exhibit strong feature learning capabilities, making them suitable for segmenting complex backgrounds and targets. However, they require large amounts of labeled data and are computationally intensive (Li et al., 2024). Clustering algorithms, as unsupervised methods, can automatically classify different regions within an image, but they have high computational complexity and struggle to accurately extract regions of interest (ROI) when leaf color distribution is uneven (Tian et al., 2019). In contrast, grayscale thresholding and color space-based segmentation methods have lower computational complexity and are well-suited for images with simple backgrounds (Xie et al., 2022; Sari and Gofuku, 2023). However, during the curing process of cigar leaves, significant morphological changes occur, which may limit the robustness of a single segmentation approach (Zhao et al., 2024b). To address this issue, this study, both color threshold segmentation and Otsu’s algorithm are employed for image segmentation (**Figure 2**). Initially, the RGB images of the front and rear surfaces of the cigar leaves are adjusted to meet the minimum and maximum color thresholds, *T_min_* and *T_max_*, respectively. Subsequently, using Equation 2, a binary image, *BI_HSV_*, is generated via the color threshold segmentation method. The original RGB image is subsequently transformed into a grayscale image, which is then smoothed via median filtering. The grayscale image is further processed via Otsu’s algorithm to generate *BI_OTSU_*. Finally, a complete binary image mask, *BI*, is created by performing logical ‘or’ operations on *BI_HSV_* and *BI_OTSU_*. This mask is then combined with the original RGB image to define the ROI in the tobacco leaf image.

(2) $$\text{grayscale=}\{\begin{array}{c} {255,\text{T}}_{\text{min}}\leq \text{[H,S,V]}\leq {\text{T}}_{\text{max}}\\ 0,\text{ others} \end{array}$$

where *T_min_* =[*H_min_*, *S_min_*, *V_min_*]=[15, 20, 15] and *T_max_* =[*H_max_*, *S_max_*, *V_max_*]=[175, 255, 255].

For the ROI of the cigar leaves’ front and rear surfaces obtained previously, color and texture features are extracted via the grey level cooccurrence matrix (GLCM) (Wang et al., 2012; Orka et al., 2024). Cigar leaves from various air-curing periods and leaf positions are converted into discrete variables through label encoding. The 36 extracted features and their descriptive statistics are presented in **Table 2** and **Supplementary Table S1**.

**Table 2:** Characteristic variables and their abbreviations.

	Feature name	Front surface feature (abbreviation)	Rear surface feature (abbreviation)
Color features	mean value of B component	*B_f_*	*B_r_*
	mean value of G component	*G_f_*	*G_r_*
	mean value of R component	*R_f_*	*R_r_*
	standard deviation of B component	*StdB_f_*	*StdB_r_*
	standard deviation of G component	*StdG_f_*	*StdG_r_*
	standard deviation of R component	*StdR_f_*	*StdR_r_*
	mean value of L* component	L_f_^*^	L_r_^*^
	mean value of a* component	a_f_^*^	a_r_^*^
	mean value of b* component	b_f_^*^	b_r_^*^
	standard deviation of L* component	StdL_f_^*^	StdL_r_^*^
	standard deviation of a* component	Stda_f_^*^	Stda_r_^*^
	standard deviation of b* component	Stdb_f_^*^	Stdb_r_^*^
Texture features	angular second moment	*ASM_f_*	*ASM_r_*
	contrast	*CON_f_*	*CON_r_*
	correlation	*Corr_f_*	*Corr_r_*
	entropy	*Entr_f_*	*Entr_r_*
	inverse difference moment	*IDM_f_*	*IDM_r_*
Leaf features	leaf position	*LP*	
	airing period	*AP*	

The image features of the front and rear surfaces of the cigar leaf are represented by subscripts *f* and *r*, respectively.

### 2.3 Image feature selection and data processing

#### 2.3.1 Image feature selection

Effective image features are essential for accurately predicting the moisture content of cigar leaves. Removing feature variables with low correlation and redundancy relative to the target variables reduces computational costs and improves the model’s generalizability (Zhao et al., 2022a). Embedding, packing and filtering are typical feature selection techniques (Xu et al., 2024). Unlike the first two methods, the filter method is a commonly used feature selection approach that is independent of specific machine learning algorithms and can identify key features before model training (Li et al., 2023). Given the large number of features in cigar leaf images from both two orientations and the strong collinearity among them, this study employs PCC for a two-step feature selection process on both front and back images of the cigar leaves. First, the correlation between image features of cigar leaves’ front and rear surfaces and their moisture content, and the results were ranked by the absolute values of the coefficients. Second, the correlations among the remaining features were subsequently analyzed, and those with high autocorrelation, as indicated by their coefficients with the moisture content, were selected. The PCC was calculated using Equation 3.


(3) $$\text{PCC}=\frac{\sum_{\text{i}=1}^{\text{n}}\left({\text{x}}_{\text{i}}-\bar{{\text{x}}_{\text{i}}}\right)\left({\text{y}}_{\text{i}}-\bar{{\text{y}}_{\text{i}}}\right)}{\sqrt{\sum_{\text{i}=1}^{\text{n}}{\left({\text{x}}_{\text{i}}-\bar{{\text{x}}_{\text{i}}}\right)}^{2}{\left({\text{y}}_{\text{i}}-\bar{{\text{y}}_{\text{i}}}\right)}^{2}}}$$

where *x_i_* and *y_i_* are both one-dimensional feature vectors, which represent the *i* ^th^ element in vectors *x* and *y*. $\bar{{\text{x}}_{\text{i}}}$ and $\bar{{\text{y}}_{\text{i}}}$ represent the mean values of two vectors.

#### 2.3.2 Data partition

To ensure data distribution consistency between the training and testing sets, the samples are divided into 5 airing periods. For each air-curing period, 35 samples are allocated to the test set, while the remainder constitute the training set. Descriptive statistics for the moisture content of the tobacco leaves in the training and testing sets are presented in **Table 3**. The small differences in the means and standard deviations between the training and testing sets, along with their similar data distributions, indicate effective data segmentation.

**Table 3:** Descriptive statistics of cigar leaves moisture content during the air-curing process.

Data set		Number	Max (%)	Min (%)	Ave (%)	Std (%)
Wilting period	total	164	92.87	81.70	86.73	2.80
	train set	129	92.87	81.70	86.65	2.73
	test set	35	92.47	82.25	87.05	3.04
Yellowing period	total	172	81.64	60.23	73.54	6.31
	train set	137	81.64	61.26	73.84	6.05
	test set	35	81.22	60.23	72.36	7.20
Browning period	total	177	60.14	27.78	42.78	10.27
	train set	142	60.14	27.78	42.80	10.32
	test set	35	59.13	27.91	42.69	10.19
Fixation period	total	211	27.72	18.03	21.91	2.79
	train set	176	27.72	18.03	21.92	2.75
	test set	35	27.47	18.18	21.90	3.03
Dry tendon period	total	156	18.00	15.15	16.59	0.79
	train set	121	18.00	15.15	16.62	0.78
	test set	35	17.86	15.15	16.47	0.83

#### 2.3.3 Data processing

Since the range and distribution of feature indices vary, standardizing data to eliminate dimensional influence can enhance the model’s prediction accuracy (Behera et al., 2021). In this study, the data were standardized to a normal distribution with a mean of 0 and a standard deviation of 1 using Equation 4.


(4) $$z=\frac{x_{i}-\bar{x}}{std}$$

where *x_i_* and $\bar{x}\;$ represent the original value and mean value of feature *x*, respectively, and where std represents the standard deviation of feature *x*.

#### 2.3.4 Leaf feature transformation

As a high-stalk crop, the top leaves of tobacco plants receive more sunlight and nutrient during growth (Tian et al., 2023), leading to varying differences in quality across leaf positions. Moreover, the apparent morphology and moisture content of cigar leaves vary significantly across different air-curing periods (**Tables 1**, **3**). Consequently, the discrete variables ‘leaf position’ and ‘airing period’ are encoded and combined with image features for use as model inputs (Hou et al., 2021).

### 2.4 Model design

Since a single model may capture only limited data insights, stacking ensemble learning combines multiple model predictions to achieve a more comprehensive view, thereby increasing the overall prediction accuracy (Liu et al., 2024). The steps for constructing the stacking ensemble learning model in this study, as illustrated in **Supplementary Figure S2**, are as follows: 1) The stacking ensemble learning model features a two-layer structure. The first layer comprises *n* machine learning algorithms optimized by a genetic algorithm, with training for the training set completed via 5-fold cross-validation. 2) The outputs from the nth base learner’s cross-validation, denoted by *v_n_*_-1_ to *v_n_*_-5_, create a new feature set. 3) This feature set serves as the training input, with the moisture content of the cigar leaves as the output for training the second-layer meta-learner. 4) The testing process mirrors the training process. Test set data are input into the first layer for prediction, yielding features *t*_1_ to *t_n_*. These features are then input into the second layer to finalize the moisture content prediction of the cigar leaves.

In this study, we evaluated the performance of the stacking ensemble learning model via five evaluation indicators of the regression model: determination coefficient (*R* ^2^), mean square error (MSE), mean absolute error (MAE), mean absolute percentage error (MAPE), and explained variance score (EVS). The specific calculation formulas are as follows Equations 5–9.


(5) $$R^{2}=1-\frac{\sum_{i=1}^{n}{\left({\hat{y}}_{i}-y_{i}\right)}^{2}}{\sum_{i=1}^{n}{\left(\bar{y}-y_{i}\right)}^{2}}$$

(6) $$\text{MSE=}\frac{1}{\text{n}}\sum_{\text{i}=1}^{\text{n}}{\left(\hat{{\text{y}}_{\text{i}}}{\text{-y}}_{\text{i}}\right)}^{2}$$

(7) $$\text{MAE=}\frac{1}{\text{n}}\sum_{\text{i}=1}^{\text{n}}\left|\hat{{\text{y}}_{\text{i}}}{\text{-y}}_{\text{i}}\right|$$

(8) $$\text{MAPE=}\frac{1}{\text{n}}\sum_{\text{i}=1}^{\text{n}}\left|\frac{\hat{{\text{y}}_{\text{i}}}{\text{-y}}_{\text{i}}}{{\text{y}}_{\text{i}}}\right|$$

(9) $$\text{EVS}=1-\frac{\sum_{i=1}^{n}{\left({\hat{y}}_{i}-\bar{y}\right)}^{2}}{\sum_{i=1}^{n}{\left(y_{i}-\bar{y}\right)}^{2}}$$

Where *n* represents the number of samples. *i*, *y_i_* represents the prediction result of the model for the *i* ^th^ sample and its true value and represents the mean value of the prediction variable.

Additionally, to assess the model’s generalizability and complexity during cross-validation, we calculated the standard deviation (Std), coefficient of variation (cv), and running time for the five metrics across 5-fold cross-validation. Given the multitude of evaluation metrics, an entropy weighting algorithm was employed to derive a comprehensive score for the model, using the mean, standard deviation (Std), and coefficient of variation (cv) of each metric as evaluation variables. The calculation method is detailed in reference (Yang et al., 2023a).

### 2.5 SHAP

The SHAP method, which is based on cooperative game theory, is an additive interpretation framework for quantifying the positive and negative impacts of each input variable on model predictions via Shapley values, providing both global and local explanations of the model’s decision-making process (El Bilali et al., 2023). Assuming that *m* samples each contain n features, the *j* ^th^ feature of the *i* ^th^ sample is represented by *x_ij_*, and its marginal contribution to the prediction result is denoted by *c_ij_*. The global contribution of a feature is represented by its weight, *w_j_*. The degree of contribution of *x_ij_* to the model’s prediction, *f*(*x_ij_*), is calculated as Equation 10.


(10) $$f\left(x_{ij}\right)=\sum_{j}^{n}c_{ij}w_{j}$$

*f*(*x_ij_*) is the contribution of *x_ij_* to the prediction result *y_i_* of the *i* ^th^ sample relative to the target output mean *f*(*x_ij_*), and the expression is as Equation 11.


(11) $$y_{i}=\bar{y}+\sum_{i=1}^{m}f\left(x_{ij}\right)$$

When *f*(*x_ij_*) ≥ 0, the impact on the model’s predictions is positive, whereas when *f*(*x_ij_*)< 0, the impact is negative. In this study, feature contributions to samples are visualized via a bee swarm plot. Given that feature influences can vary across samples, we employ the relative average absolute Shapley value, *RV_j._* The calculation formula is as Equation 12.


(12) $$RV_{j}=\left(\frac{1}{m}\sum_{i=1}^{m}\left|f\left(x_{ij}\right)\right|/\sum_{j=1}^{n}\frac{1}{m}\sum_{i=1}^{m}\left|f\left(x_{ij}\right)\right|\right)\times 100\%$$

## 3 Results

### 3.1 Image feature selection

**Figure 3:**
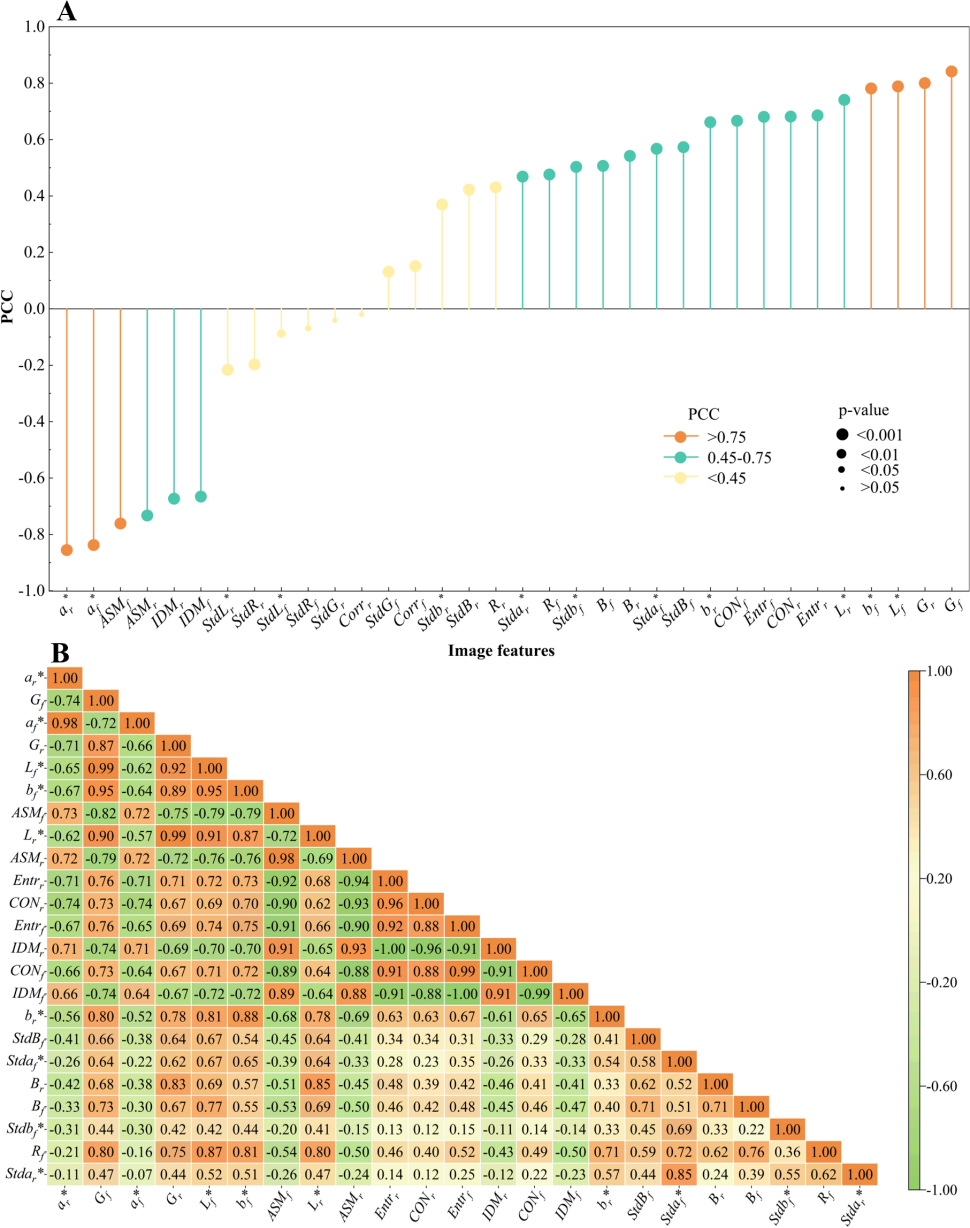
Image feature and cigar leaves moisture content correlation analysis **(A)** Correlation between image features and moisture content of cigar leaves. **(B)** The autocorrelation between image features.

The correlations between the extracted image features of cigar leaves and their moisture content are calculated and ranked. These variables are categorized into three groups according to the absolute value of the Pearson correlation coefficient (|PCC|) between the feature variables and moisture content. The feature variables with |PCC|>0.75 are defined as ‘important features’, the feature variables with 0.75>|PCC|>0.45 are defined as ‘relatively important features’, and the feature variables with 0.45>|PCC| are defined as ‘unimportant features’ (**Figure 3A**). To reduce feature redundancy, ‘unimportant features’ (*R_r_*, *StdB_r_*, Stdb_r_^*^, *Corr_f_*, *StdG_f_*, *Corr_r_*, *StdG_r_*, *StdR_f_*, StdL_f_^*^, *StdR_r_*, and StdL_f_^*^) are removed. Only ‘important features’ and ‘relatively important features’ are retained for further analysis.

To avoid the influence of the autocorrelation between image features on the model performance, the |PCC| calculated according to **Figure 3A** is sorted, and the autocorrelation between features is calculated (**Figure 3B**). As the correlation with moisture content decreases, the autocorrelation among image features similarly diminishes, moving from the upper left to the lower right in the figure. Among the ‘important features’ (a_r_^*^, *G_f_*, a_f_^*^, *G_r_*, L_f_^*^, *b_f_* ^*^, and *ASM_f_*), a_r_^*^, L_f_^*^, and *b_f_* ^*^ are strongly correlated with other features and are therefore removed. For the texture features, *ASM_f_* is the only ‘important feature’. This feature is highly correlated with other texture features but weakly correlated with color features. Consequently, *ASM_f_*, which has a high correlation with moisture content, and *IDM_f_*, which has the weakest correlation with other features, are retained. The remaining features are retained because of their low intercorrelation. Ultimately, the optimal feature subset retained includes *B_f_*, *G_f_*, *R_f_*, *StdB_f_*, a_f_^*^, *Stda_f_* ^*^, *Stdb_f_* ^*^, *ASM_f_*, *IDM_f_*, *B_r_*, *G_r_*, *b_r_* ^*^, *Stda_r_* ^*^, *LP*, and *AP.*

### 3.2 Model selection

**Figure 4:**
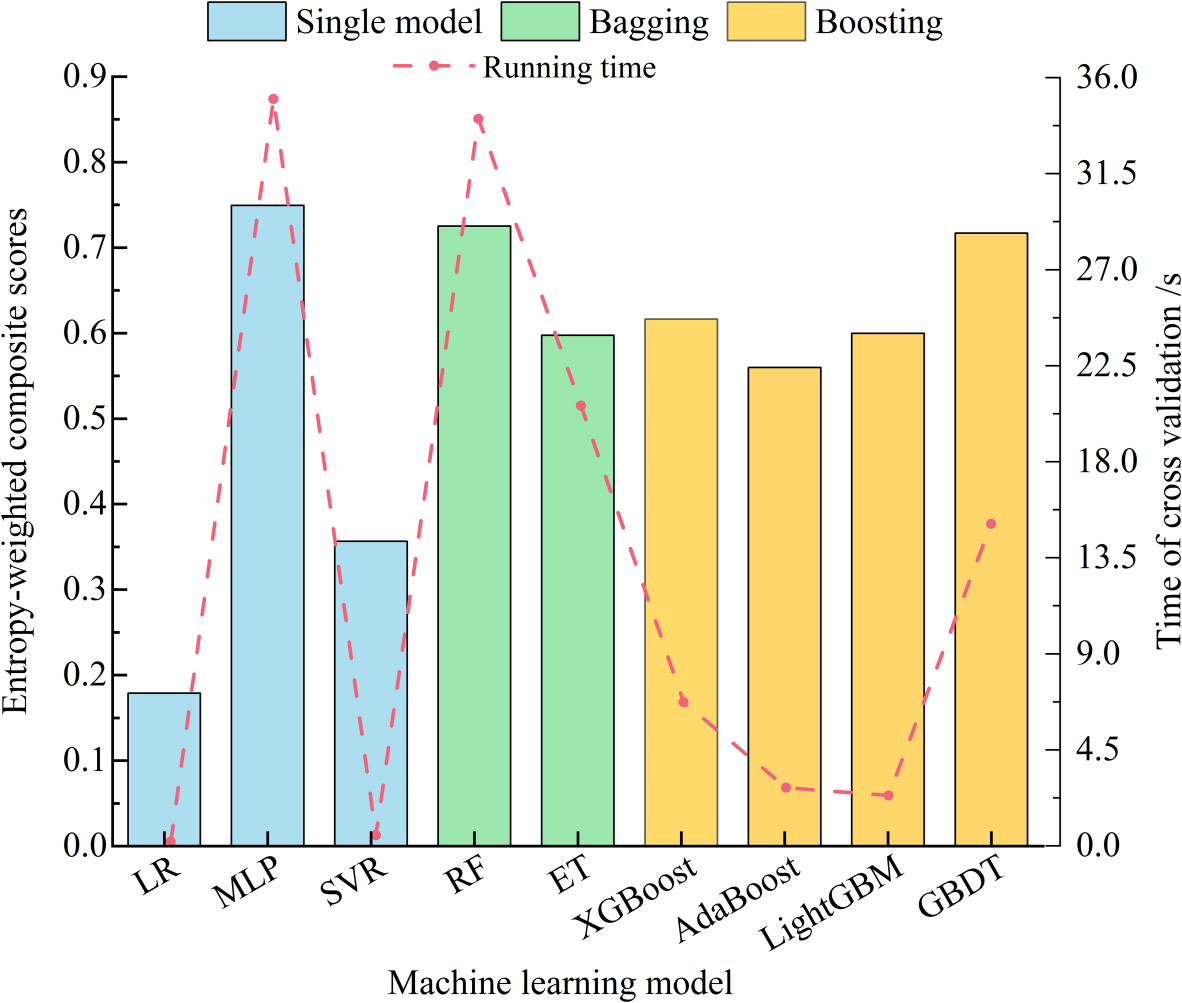
Entropy-weighted scores and running time of 5-fold cross-validation of each candidate base model.

Three single models (LR, SVR, and MLP), two bagging models (RF and ET), and four boosting models (GBDT, XGBoost, AdaBoost, and LightGBM) are selected as candidate machine learning models. The genetic algorithm (Abba et al., 2023) was employed for optimize the hyperparameters of 9 machine learning models. The optimized hyperparameters for each model are provided in **Supplementary Table S2**, The optimal image feature subset subsequently serves as the input for each candidate model, which is evaluated via 5-fold cross-validation. Model prediction performance is assessed via the mean values of the five-evaluation metrics (*R* ^2^_mean_, MSE_mean_, MAE_mean_, MAPE_mean_, EVS_mean_). Additionally, the standard deviation (*R* ^2^_Std_, MSE_Std_, MAE_Std_, MAPE_Std_, EVS_Std_) and coefficient of variation (*R* ^2^_cv_, MSE_cv_, MAE_cv_, MAPE_cv_, EVS_cv_) for each of the evaluation indicators are calculated via 5-fold cross-validation to assess each model’s generalizability (**Supplementary Table S3**), resulting in a total of 15 indicators. Given the large number of evaluation metrics, *R* ^2^_mean_ and EVS_mean_ are designated positive indicators, whereas the remaining 13 are considered negative indicators. The comprehensive score for each model is calculated via the entropy weight method (Cao et al., 2024), with the 5-fold cross-validation running time reflecting the model complexity. The results are shown in **Figure 4**. For the stacking ensemble learning model, the base learner should be as diverse as possible to enhance the model performance (Jiang et al., 2023); thus, MLP, RF, and GBDT are selected as the best base learners. For the meta-learner, a less complex model with relatively superior performance is preferred. Hence, among the single models, LR is selected as the meta-learner because it has the shortest cross-validation time.

### 3.3 Prediction of moisture content during cigar leaf air-curing

**Figure 5:**
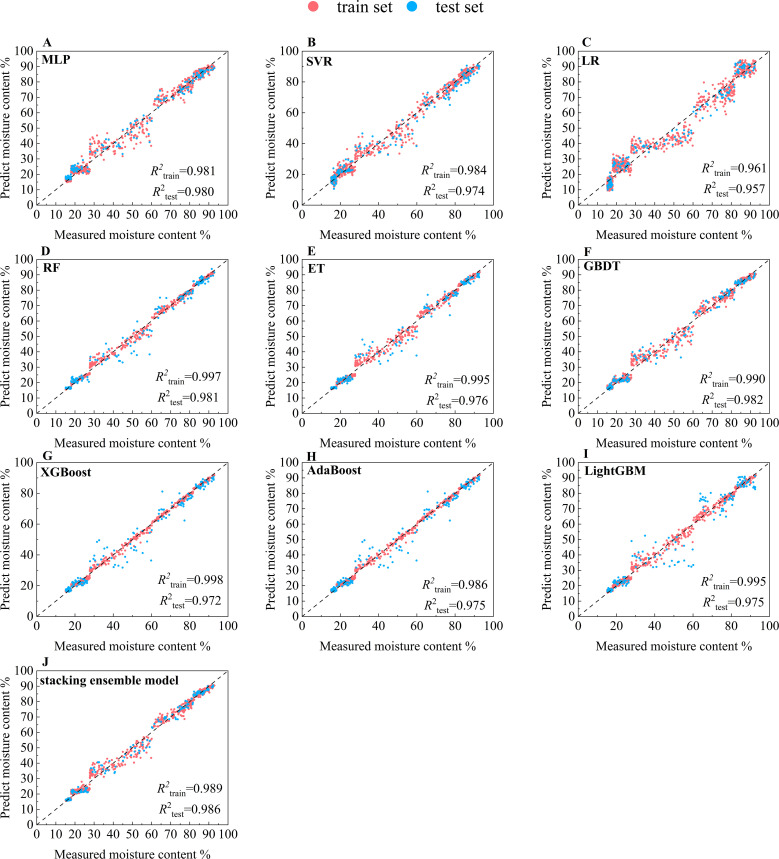
Prediction results of the moisture content of cigar leaves during the air-curing process by different model. **(A)** MLP. **(B)** SVR. **(C)** LR. **(D)** RF. **(E)** ET. **(F)** GBDT. **(G)** XGBoost. **(H)** AdaBoost. **(I)** LightGBM. **(J)** stacking ensemble model. The black dashed line represents a 1:1 relationship, where red indicates the train set and blue indicates the test set.

To explore each model’s ability to predict the moisture content of cigar leaves during the air-curing process, the testing and training sets were analyzed separately. Additionally, to validate the performance of the stacking ensemble learning model developed in this study for predicting the cigar leaf moisture content, the prediction results of each base models were compared, **Figure 5** and **Supplementary Figure S3** shows the prediction results of different models. It can be seen that among the three models, MLP, RF and GBDT have the best prediction effect, achieving *R* ^2^_test_ values of 0.980~0.982. The stacking ensemble model constructed in this study has the best prediction effect on the test set, achieving *R* ^2^_test_ values of 0.989. Further observation revealed that when the moisture content of cigar leaf ranged between 30% and 60%, the prediction performance of each base model for the test set is poor, while the stacking ensemble learning model overcomes this weakness. After entering the browning period, until the cigar leaves enter the dry tendon period, the moisture of cigar leaves is not evenly lost, which leads to differences in the changes of color and texture features of different leaves. It is difficult to fully capture the complex and non-linear pattern changes of the image only by a single model. However, the stacking ensemble learning model can learn the non-linear relationship between the prediction results of the base model, so as to better capture the complex feature changes of the apparent morphology of cigar leaves in this interval.

### 3.4 Feature importance analysis

**Figure 6:**
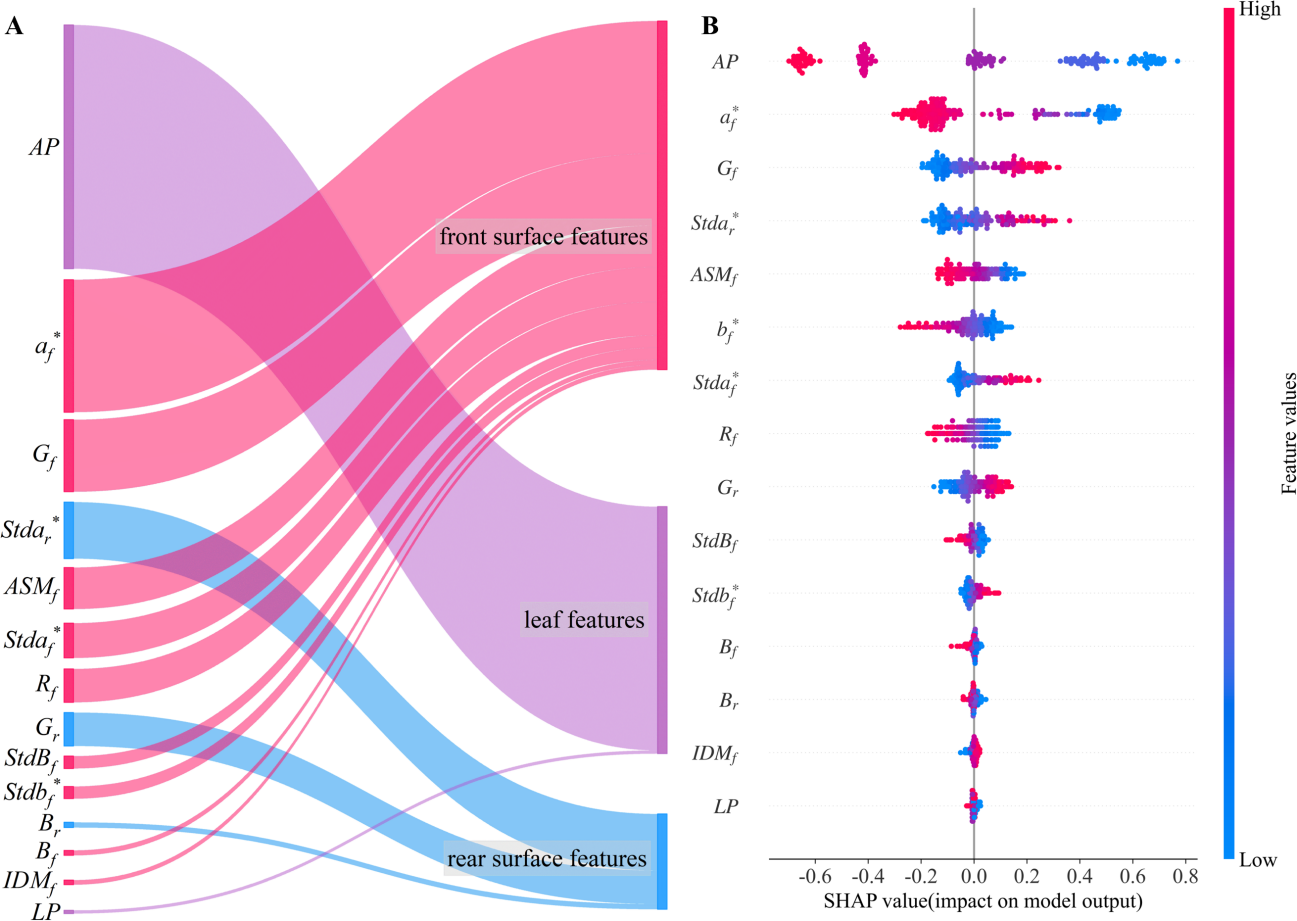
Analysis of feature importance. **(A)** contribution of the three types of features. **(B)** Bee swarm plot of each feature. *AP* and *LP* are discrete variables, and blue to red represents cigar leaves from the wilting period to the dry tendon period and from the lower leaves to the upper leaves, respectively.

To explore the key characteristics of moisture content prediction during the cigar leaf air-curing process, the SHAP method is employed to elucidate the prediction results of the stacking ensemble learning model. The input variables are categorized into three groups on the basis of feature type: front surface features, rear surface features, and leaf features. The relative contribution of each feature to the prediction results is calculated on the basis of its average absolute SHAP value (**Figure 6A**). The relative contribution of each feature category to the prediction of the cigar leaf moisture content is generally as follows: front surface features (45.5%), leaf features (38.5%), and rear surface features (17.0%). Input variables such as *AP*, a_f_^*^, *G_f_*, Stda_r_^*^, b_f_^*^, and *ASM_f_* significantly contribute to the model’s prediction accuracy. In order to deeply analyze the decision-making mechanism of the model for predicting the moisture content of tobacco leaves, we use the bee colony diagram to show the SHAP value distribution of different input features, in which the features are sorted by importance. The bee swarm plot illustrates how different feature values from the test set contribute to the model’s prediction results (**Figure 6B**). For each input features, *AP* positively influences model predictions during the wilting and yellowing periods but negatively influences them during the fixation period. Additionally, the green component (*G_f_*, *G_r_*) and the standard deviation of the a* component (*Stda_f_* ^*^, *Stda_r_* ^*^) influence the prediction results similarly to the *AP*, in contrast to the a* and b* components and the angular second moment (a_f_^*^, b_f_^*^, and *ASM_f_*) of the front surface images. This occurs as the cigar leaves undergo air-curing, transitioning from green to brown. During the fixation period, the leaf color becomes more uniform, and the grey distribution stabilizes.

### 3.5 Interaction between the airing period and image features

**Figure 7:**
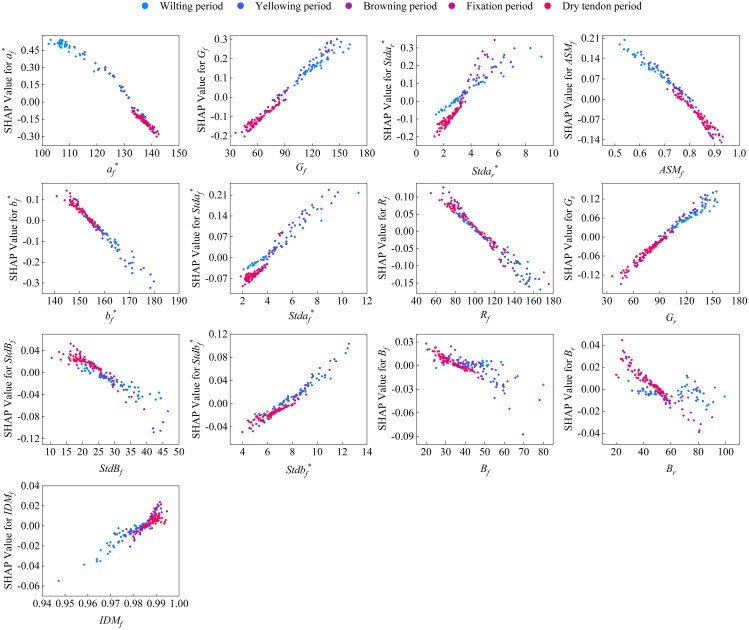
Partial dependence plot (PDP) of input image features and the *AP*.

The discrete variable *AP* represents the five periods experienced by the cigar leaves during the air-curing process. With the change of the air-curing period, the apparent morphology of the tobacco leaves also changes. The feature importance ranking revealed that *AP* had the greatest impact on predicting the moisture content of cigar leaves. Interestingly, the influence of this feature on the model predictions shifted from positive to negative as the air-curing process progressed (**Figure 6**). To further investigate the interactions between input features, **Figure 7** illustrates the SHAP interaction effects between the discrete variable *AP* (airing period) and various leaf image features. When *AP* changes, if two features exhibit a similar trend in their SHAP values, it suggests that they have a synergistic contribution to the model’s predictive outcome. As observed in the figure, with the progression of the air-curing process, the contributions of a_f_^*^, *G_f_*, *ASM_f_*, and *G_r_* to the model gradually decrease, whereas b_f_^*^ and *R_f_* exhibit an opposite pattern. Additionally, although Stda_r_^*^ and Stda_f_^*^ demonstrate relatively weak interaction effects with *AP*, their SHAP values follow a similar trend across different airing period, indicating that they may have a consistent influence on model predictions at specific phases.

## 4 Discussion

### 4.1 Prediction results under different feature combinations

**Figure 8:**
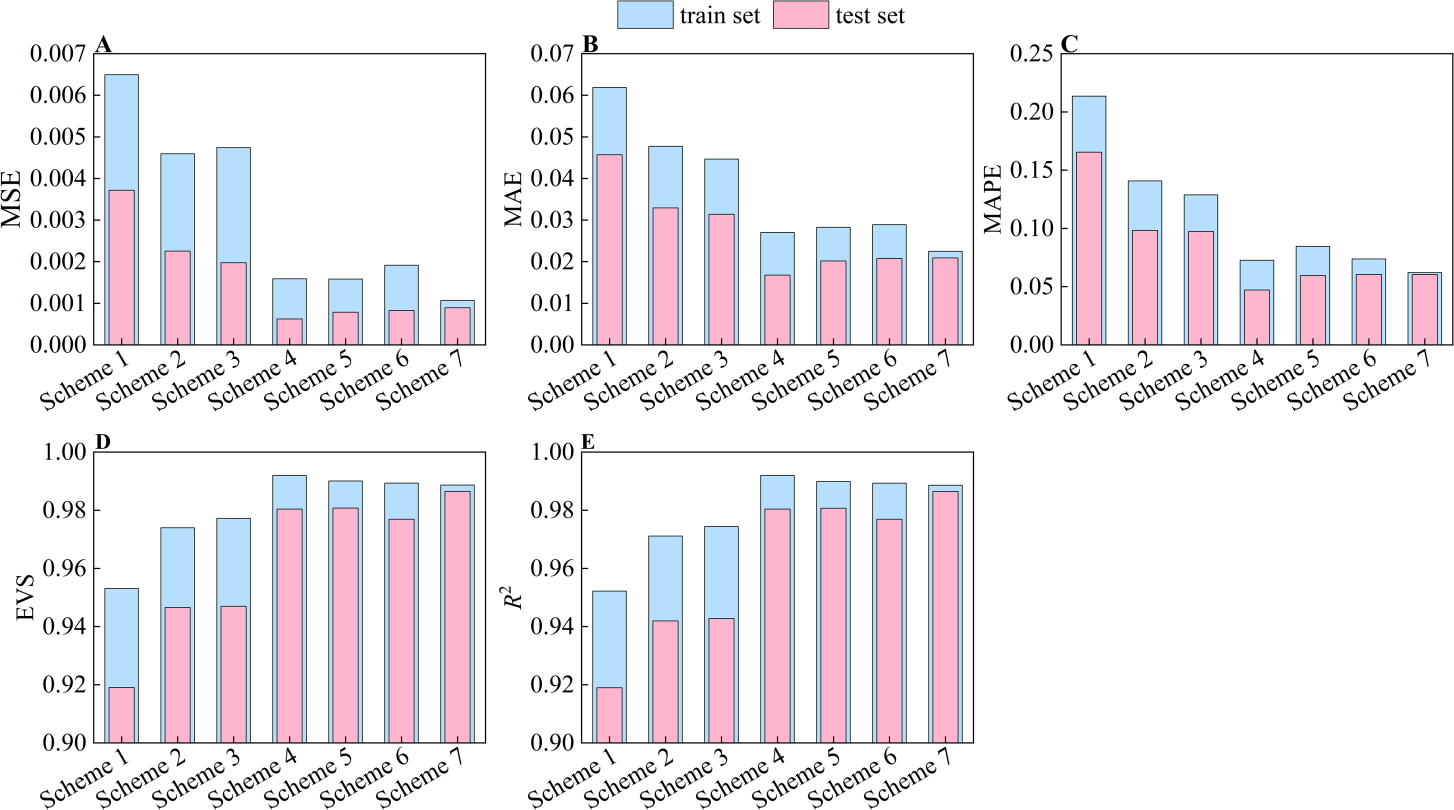
Model performance under different feature combinations. **(A)** MSE. **(B)** MAE. **(C)** MAPE. **(D)** EVS. **(E)** *R* ^2^. Scheme 1: front surface features; Scheme 2: rear surface features; Scheme 3: front and rear surface features; Scheme 4: front surface features and leaf features; Scheme 5: rear surface features and leaf features; Scheme 6: original features; and Scheme 7: optimized feature subset.

As the cigar leaves lose moisture while hanging in the drying room, they gradually shrunk (Zhao et al., 2022b). In previous studies, only the front images of flattened cigar leaves were captured. Thus, their real shape during the air-curing process was not accurately represented. A significant contribution of this study is the development of a method that predicts the moisture content of cigar leaves during air-curing by utilizing image information from two orientations and characteristics of the leaves in their natural suspended state. To demonstrate the superiority of the preferred feature subset constructed in this study, we considered three types of features: front surface features, rear surface features, and leaf features. Seven feature combination schemes were developed: Scheme 1: front surface features; Scheme 2: rear surface features; Scheme 3: front and rear surface features; Scheme 4: front surface features and leaf features; Scheme 5: rear surface features and leaf features; Scheme 6: original features; and Scheme 7: optimized feature subset. The prediction results for each of the seven schemes are displayed in **Figure 8** Model performance under different feature combinations. The model has lower prediction accuracy when only image features are used as input (Schemes 1-3). Combining image and leaf features (Schemes 4-6) significantly improves the model’s prediction accuracy. However, compared with that using Scheme 7, the model’s generalizability is worse with the other schemes. An increase in the number of input variables can degrade the model performance due to collinearity among features. The image features of the front and rear surfaces of the cigar leaves, as extracted in this study, exhibit a strong correlation with the target variables and weak autocorrelation. Using all the features as model inputs leads to overfitting due to feature redundancy.

### 4.2 Further analysis of the stacking strategy

**Figure 9:**
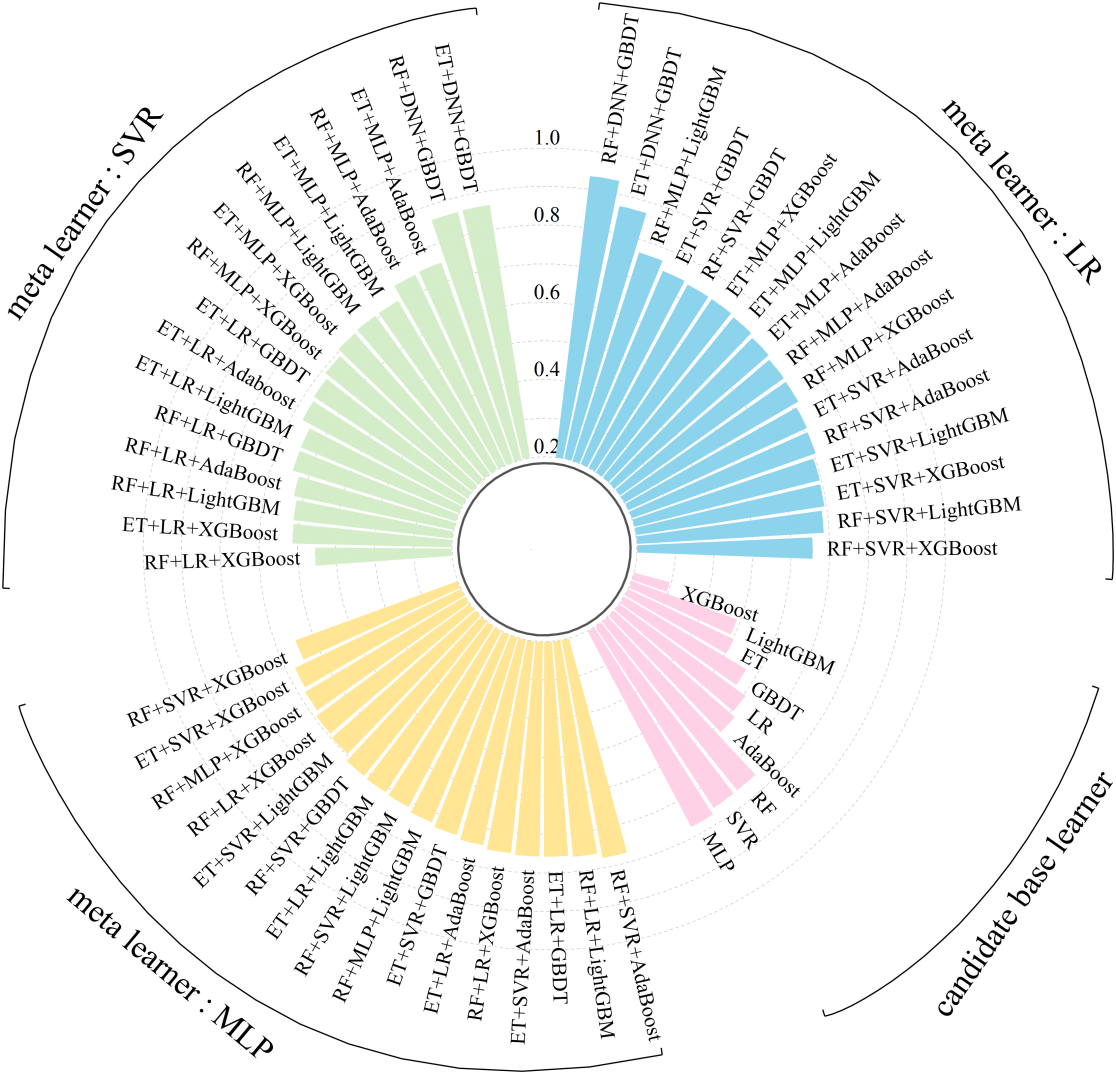
Comprehensive scores of different stacking strategy.

Machine learning models vary in performance across different feature spaces. The stacking ensemble learning model enhances prediction accuracy and reduces errors and deviations by synthesizing the outputs of each base learner and leveraging their advantages. In this study, the entropy weight method is used to assess the performance and generalizability of candidate base learners during 5-fold cross-validation. The highest-scoring models from three categories (single, bagging, and boosting models) are chosen as the base learners for constructing the stacking ensemble learning model. To demonstrate the superiority of our constructed model, nine candidate base models were utilized to create various stacking ensemble learning model configurations. To assess each configuration’s performance and robustness, five metrics (EVS, *R* ^2^, MSE, MAE, and MAPE) were used to evaluate the prediction outcomes and their variance between the train and test sets. The comprehensive scores for each metric were computed via the entropy weight method (**Figure 9**, **Supplementary Table S4**). Overall, across all configurations, the stacking ensemble model developed in this study, with RF, MLP, and GBDT as the base learners and LR as the meta-learner, achieved the highest comprehensive score. Among the candidate base learners, MLP scored the highest at 0.774. For the three types of ensemble model schemes composed of different meta-learners, significant performance variability was noted when LR and SVR served as meta-learners. In contrast, there were minimal performance differences when using configurations with MLP as the meta-learner. During the construction of the stacking ensemble model, meta-learners primarily fit the predictions from base learners. Complex meta-learners, however, increase both the computational cost and the risk of overfitting (van der Laan et al., 2007).

### 4.3 Comparison with related studies

To comprehensively evaluate the superiority of the model proposed in this study, we compared its prediction results with those from other studies on moisture content prediction during cigar leaf air-curing process. Specifically, we compared our results with those of Yang et al. (2023b) and our previous study (Xing et al., 2024), as presented in **Table 4**, the results indicate that our model outperforms both studies, demonstrating that the proposed stacking ensemble model effectively leverages image information from both the front and rear surfaces of cigar leaves during air-curing stage. Moreover, in the aforementioned studies, images of the cigar leaves were captured after manually flattening them, a process that is relatively complex and fails to accurately reflect the actual state of the leaves during curing. In contrast, our approach eliminates this limitation, further enhancing the reliability of moisture content predictions. In summary, the proposed method has been proven effective for predicting the moisture content of cigar tobacco leaves during the curing process.

**Table 4:** Evaluation indicators for cigar leaf moisture content prediction.

Related studies	Image type	Model	*R* ^2^	MSE
Xing et al. (2024)	RGB image of cigar leaves in flat state	RF	0.980	0.001
Yang et al. (2023b)	RGB image of cigar leaves in flat state	Convolutional neural networks (CNN)	0.904	0.006
This study	Front and back image of cigar leaves in suspend state	Stacking ensemble model	0.986	0.001

### 4.4 Limitations and future work

Although the stacking ensemble model constructed in this study can accurately predict the moisture content of cigar leaves during air-curing process, there are still some limitations in this study. First, the data source is single, covering only a single producing area and variety. However, in actual production, factors such as climatic conditions in different areas and the variety characteristics of different cigar leaves will affect the water loss rate and morphological changes of cigar leaves during the air-curing process. Future research needs to collect samples under various production conditions to ensure the wide applicability of the model. Second, In the process of image acquisition, the change of external illumination and leaf positioning error will still affect the stability of the image feature extraction, which will affect the final output of the model. In future research, deep learning methods of adaptive feature learning, such as residual network (ResNet) and vision Transformer (ViT), should be introduced and data sets should be expanded through data enhancement methods to improve the robustness of the model and reduce the impact of external environment on feature extraction. Third, in the actual air-curing process, the change of environmental light and the limitation of equipment conditions may bring some challenges to the wide application of this research method in large-scale production environment. Therefore, future research can explore optimization strategies based on smart mobile terminals and combine lightweight deep learning models to improve the robustness and applicability of the method, so as to better meet the actual production needs.

## 5 Conclusion

Accurate monitoring of moisture content during the cigar leaves air-curing process can help technicians better grasp the status of cigar leaves to make corresponding adjustments to the airing technology in a timely manner. In this study, a moisture content prediction model was constructed for air-cured cigar leaves via a stacking ensemble learning model, which incorporates images of leaves in their natural suspended state and data on the leaf position and airing period. The SHAP method was employed to interpret the model’s prediction results. The main results are as follows:

1. Compared with each base learner alone, the stacking ensemble learning model constructed in this study can achieve higher prediction accuracy and better generalization performance (*R* ^2^_train_=0.990, *R* ^2^_test_=0.989).
2. By integrating the SHAP method, the contribution of each input feature to the model’s predictions was quantified and potential feature interactions was uncover. The results show that *AP*, *a_f_* ^*^, *G_f_*, *Stda_r_* ^*^, *b_f_* ^*^, and *ASM_f_* have a significant impact on model performance. Additionally, *a_f_* ^*^, *G_f_*, *ASM_f_*, *G_r_* exhibited a synergistic relationship, as do b_f_^*^ and *R_f_*, and Stda_r_^*^ and Stda_f_^*^.
3. The morphological changes in cigar leaves vividly reflect the internal water loss they undergo. Based on the image features of cigar leaves, the moisture content of the leaves can be accurately predicted. In summary, this research offers new insights into achieving enhanced intelligence in the air-curing process for cigar leaves.

## Data Availability

The original contributions presented in the study are included in the article/**Supplementary Material**. Further inquiries can be directed to the corresponding authors.

## References

[B1] Abba S. I. Benaafi M. Usman A. G. Aljundi I. H. (2023). Sandstone groundwater salinization modelling using physicochemical variables in Southern Saudi Arabia: Application of novel data intelligent algorithms. Ain. Shams. Eng. J. 14, 101894. doi: 10.1016/j.asej.2022.101894

[B2] Al Shamsi A. A. Abdallah S. (2023). Ensemble stacking model for sentiment analysis of Emirati and Arabic dialects. J. King Saud. Univ.-Comput. Inf. Sci. 35, 101691. doi: 10.1016/j.jksuci.2023.101691

[B3] Arevalo-Ramirez T. Villacrés J. Fuentes A. Reszka P. Auat Cheein F. A. (2020). Moisture content estimation of *Pinus radiata* and *Eucalyptus globulus* from reconstructed leaf reflectance in the SWIR region. Biosyst. Eng. 193, 187–205. doi: 10.1016/j.biosystemseng.2020.03.004

[B4] Behera S. K. Rath A. K. Sethy P. K. (2021). Maturity status classification of papaya fruits based on machine learning and transfer learning approach. Inf. Process Agric. 8, 244–250. doi: 10.1016/j.inpa.2020.05.003

[B5] Cakiroglu C. Demir S. Hakan Ozdemir M. Latif Aylak B. Sariisik G. Abualigah L. (2024). Data-driven interpretable ensemble learning methods for the prediction of wind turbine power incorporating SHAP analysis. Expert Syst. Appl. 237, 121464. doi: 10.1016/j.eswa.2023.121464

[B6] Cao W. Zhang Z. Fu Y. Zhao L. Ren Y. Nan T.. (2024). Prediction of arsenic and fluoride in groundwater of the North China Plain using enhanced stacking ensemble learning. Water Res. 259, 121848. doi: 10.1016/j.watres.2024.121848 38824797

[B7] Dong X. Yu Z. Cao W. Shi Y. Ma Q. (2020). A survey on ensemble learning. Front. Comput. Sci. 14, 241–258. doi: 10.1007/s11704-019-8208-z

[B8] El Bilali A. Abdeslam T. Ayoub N. Lamane H. Ezzaouini M. A. Elbeltagi A. (2023). An interpretable machine learning approach based on DNN, SVR, Extra Tree, and XGBoost models for predicting daily pan evaporation. J. Environ. Manage. 327, 116890. doi: 10.1016/j.jenvman.2022.116890 36459782

[B9] Fang X. Zhang J. Zhao X. Zhang L. Zhou D. Yu C.. (2024). Optimising maize threshing by integrating DEM simulation and interpretive enhanced predictive modelling. Biosyst. Eng. 244, 93–106. doi: 10.1016/j.biosystemseng.2024.06.001

[B10] Hou H. Chen X. Li M. Zhu L. Huang Y. Yu J. (2021). Prediction of user outage under typhoon disaster based on multi-algorithm Stacking integration. Int. J. Electr. Power Energy Syst. 131, 107123. doi: 10.1016/j.ijepes.2021.107123

[B11] Hou H. Liu C. Wei R. He H. Wang L. Li W. (2023). Outage duration prediction under typhoon disaster with stacking ensemble learning. Reliab. Eng. Syst. Saf. 237, 109398. doi: 10.1016/j.ress.2023.109398

[B12] Jiang H. Zhang S. Yang Z. Zhao L. Zhou Y. Zhou D. (2023). Quality classification of stored wheat based on evidence reasoning rule and stacking ensemble learning. Comput. Electron. Agric. 214, 108339. doi: 10.1016/j.compag.2023.108339

[B13] Li X. Chen M. He S. Xu X. He L. Wang L.. (2024). Estimation of soybean yield based on high-throughput phenotyping and machine learning. Front. Plant Sci. 15. doi: 10.3389/fpls.2024.1395760 PMC11187272 38903425

[B14] Li X. Xu X. Chen M. Xu M. Wang W. Liu C.. (2022). The field phenotyping platform’s next darling: Dicotyledons. Front. Plant Sci. 13. doi: 10.3389/fpls.2022.935748 PMC9449727 36092402

[B15] Li X. Xu X. Xiang S. Chen M. He S. Wang W.. (2023). Soybean leaf estimation based on RGB images and machine learning methods. Plant Methods 19, 59. doi: 10.1186/s13007-023-01023-z 37330499 PMC10276400

[B16] Li Z. (2022). Extracting spatial effects from machine learning model using local interpretation method: An example of SHAP and XGBoost. Comput. Environ. Urban Syst. 96, 101845. doi: 10.1016/j.compenvurbsys.2022.101845

[B17] Liu L. Zhao G. Liang W. Jian Z. (2024). Hybrid stacking ensemble algorithm and simulated annealing optimization for stability evaluation of underground entry-type excavations. Undergr. Space 17, 25–44. doi: 10.1016/j.undsp.2023.11.0022467-9674

[B18] Lu Y. Li T. Hu H. Zeng X. (2023). Short-term prediction of reference crop evapotranspiration based on machine learning with different decomposition methods in arid areas of China. Agric. Water Manage. 279, 108175. doi: 10.1016/j.agwat.2023.108175

[B19] Lundberg S. M. Lee S. I. (2017). A unified approach to interpreting model predictions. Adv Neur Inf. 30, 4765–4774. doi: 10.5555/3295222.3295230

[B20] Nirmaan A. M. C. Prasantha B. D. R. Peiris B. L. (2020). Comparison of microwave drying and oven-drying techniques for moisture determination of three paddy (*Oryza sativa* L.) varieties. Chem. Biol. Technol. Agric. 7, 1. doi: 10.1186/s40538-019-0164-1

[B21] Orka N. A. Haque E. Uddin M. N. Ahamed T. (2024). Nutrispace: A novel color space to enhance deep learning based early detection of cucurbits nutritional deficiency. Comput. Electron. Agric. 225, 109296. doi: 10.1016/j.compag.2024.109296

[B22] Salam R. Islam A. R. M. T. (2020). Potential of RT, bagging and RS ensemble learning algorithms for reference evapotranspiration prediction using climatic data-limited humid region in Bangladesh. J. Hydrol. 590, 125241. doi: 10.1016/j.jhydrol.2020.125241

[B23] Sari Y. A. Gofuku A. (2023). Measuring food volume from RGB-Depth image with point cloud conversion method using geometrical approach and robust ellipsoid fitting algorithm. J. Food Eng. 358, 111656. doi: 10.1016/j.jfoodeng.2023.111656

[B24] Singh S. K. Vidyarthi S. K. Tiwari R. (2020). Machine learnt image processing to predict weight and size of rice kernels. J. Food Eng. 274, 109828. doi: 10.1016/j.jfoodeng.2019.109828

[B25] Tao H. Zhou R. Tang Y. Li W. Yao X. Cheng T.. (2024). Estimating wheat spike-leaf composite indicator (SLI) dynamics by coupling spectral indices and machine learning. Crop J. 12, 927–937. doi: 10.1016/j.cj.2024.04.003

[B26] Tian K. Li J. Zeng J. Evans A. Zhang L. (2019). Segmentation of tomato leaf images based on adaptive clustering number of K-means algorithm. Comput. Electron. Agric. 165, 104962. doi: 10.1016/j.compag.2019.104962

[B27] Tian Y. Zeng Z. Gong H. Zhou Y. Qi L. Zhen W. (2023). Simulation of tensile behavior of tobacco leaf using the discrete element method (DEM). Comput. Electron. Agric. 205, 107570. doi: 10.1016/j.compag.2022.107570

[B28] van der Laan M. J. Polley E. C. Hubbard A. E. (2007). Super learner. Stat. Appl. Genet. Mol. Biol. 6, 25. doi: 10.2202/1544-6115.1309 17910531

[B29] Wang S. Liu K. Yu X. Wu D. He Y. (2012). Application of hybrid image features for fast and non-invasive classification of raisin. J. Food Eng. 109, 531–537. doi: 10.1016/j.jfoodeng.2011.10.028

[B30] Wei Y. Li X. He Y. (2021). Generalisation of tea moisture content models based on VNIR spectra subjected to fractional differential treatment. Biosyst. Eng. 205, 174–186. doi: 10.1016/j.biosystemseng.2021.03.006

[B31] Xie W. Wei S. Zheng Z. Chang Z. Yang D. (2022). Developing a stacked ensemble model for predicting the mass of fresh carrot. Postharvest Biol. Technol. 186, 111848. doi: 10.1016/j.postharvbio.2022.111848

[B32] Xing Z. Zhang K. Liu X. Ma M. Liu B. Ding S.. (2024). Prediction of moisture content in cigar tobacco leaves during the drying process based on random forest feature selection. Trans. Chin. Soc Agric. Eng. Trans. CSAE 40, 343–354. doi: 10.11975/j.Issn.1002-6819.202311082

[B33] Xu W. Song C. Li Z. Song F. Hu S. Li J.. (2018). Temperature gradient control during microwave combined with hot air drying. Biosyst. Eng. 169, 175–187. doi: 10.1016/j.biosystemseng.2018.02.013

[B34] Xu Z. Yang F. Tang C. Wang H. Wang S. Sun J.. (2024). FG-HFS: A feature filter and group evolution hybrid feature selection algorithm for high-dimensional gene expression data. Expert Syst. Appl. 245, 123069. doi: 10.1016/j.eswa.2023.123069

[B35] Yan G. Zhao W. Wang C. Shi Z. Li H. Yu Z.. (2024). A comparative study of machine learning models for respiration rate prediction in dairy cows: Exploring algorithms, feature engineering, and model interpretation. Biosyst. Eng. 239, 207–230. doi: 10.1016/j.biosystemseng.2024.01.010

[B36] Yang H. Tong Z. Yang W. Hua X. Liu X. Zhang H.. (2023b). Moisture content monitoring of cigar leaves during drying based on a Convolutional Neural Network. Int. Agrophysics 37, 225–234. doi: 10.31545/intagr/165775

[B37] Yang B. Wu X. Hao J. Xu D. Liu T. Xie Q. (2023a). Estimation of wood failure percentage under shear stress in bamboo-wood composite bonded by adhesive using a deep learning and entropy weight method. Ind. Crops Prod. 197, 116617. doi: 10.1016/j.indcrop.2023.116617

[B38] Yang J. Xue F. Li D. Chen J. Shi G. Song G.. (2024). Oxygen regulation of microbial communities and chemical compounds in cigar tobacco curing. Front. Microbiol. 15. doi: 10.3389/fmicb.2024.1425553 PMC11300322 39109208

[B39] Zhao S. Li Y. Liu F. Song Z. Yang W. Lei Y.. (2024b). Dynamic changes in fungal communities and functions in different air-curing stages of cigar tobacco leaves. Front. Microbiol. 15. doi: 10.3389/fmicb.2024.1361649 PMC10985334 38567079

[B40] Zhao P. Wang S. Duan S. Wang A. Meng L. Hu Y. (2024a). TCSRNet: a lightweight tobacco leaf curing stage recognition network model. Front. Plant Sci. 15. doi: 10.3389/fpls.2024.1474731 PMC11688197 39744605

[B41] Zhao S. Wang M. Ma S. Cui Q. (2022a). A feature selection method via relevant-redundant weight. Expert Syst. Appl. 207, 117923. doi: 10.1016/j.eswa.2022.117923

[B42] Zhao S. Wu Z. Lai M. Zhao M. Lin B. (2022b). Determination of optimum humidity for air-curing of cigar tobacco leaves during the browning period. Ind. Crops Prod. 183, 114939. doi: 10.1016/j.indcrop.2022.114939

