# Supplementary material for: Moisture content prediction of cigar leaves air-curing process based on stacking ensemble learning model
Source: Front Plant Sci. 2025 Mar 26;16:1553110. doi: 10.3389/fpls.2025.1553110 (PMC11979227; doi:10.3389/fpls.2025.1553110)
Supplement: Supplementary file 1 [file DataSheet1.docx]

**
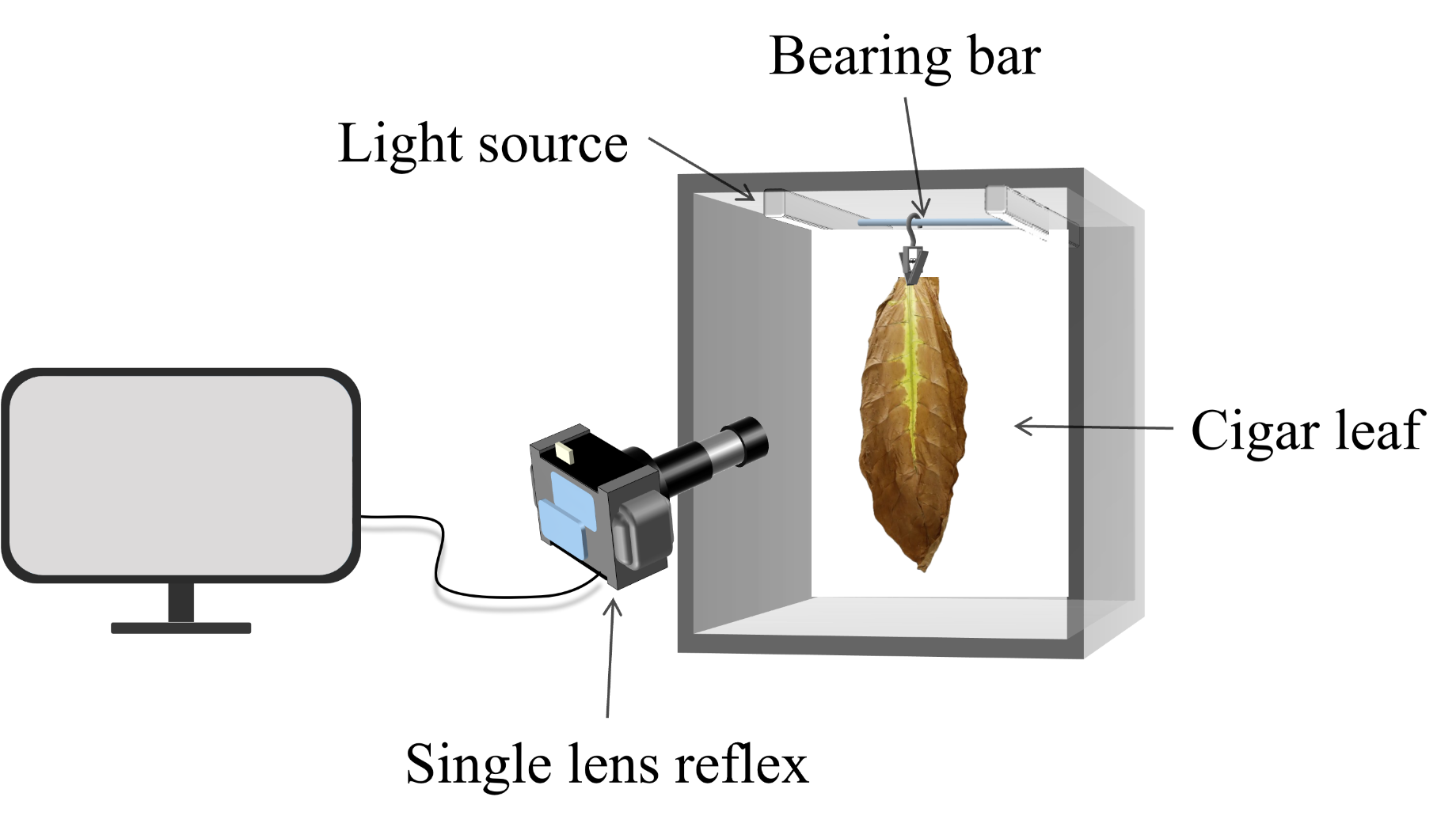
**

**Supplementary Figure 1.**

The image acquisition device.

**Supplementary Table S1.**

Descriptive statistics of cigar leaf characteristic variables

|  |  | | max | min | mean | std |  |  | max | min | mean | std |
| --- | --- | --- | --- | --- | --- | --- | --- | --- | --- | --- | --- | --- |
| Front surface features | *B*f | | 86.73 | 18.84 | 39.39 | 11.02 | Rear surface features | *B_r_* | 101.69 | 20.05 | 51.97 | 17.58 |
|  | *G*f | | 167.65 | 32.86 | 88.67 | 33.18 |  | *G_r_* | 161.55 | 36.03 | 97.02 | 30.15 |
|  | *R*f | | 176.98 | 46.40 | 107.19 | 27.54 |  | *R_r_* | 168.08 | 54.86 | 113.90 | 24.61 |
|  | *StdB*f | | 59.13 | 10.46 | 25.12 | 6.89 |  | *StdB_r_* | 44.44 | 11.99 | 25.09 | 5.73 |
|  | *StdG*f | | 59.95 | 15.75 | 30.05 | 6.74 |  | *StdG_r_* | 55.08 | 14.84 | 28.14 | 6.11 |
|  | *StdR*f | | 49.74 | 15.54 | 31.47 | 5.39 |  | *StdR_r_* | 47.29 | 14.45 | 28.41 | 5.28 |
|  | *L*f*^*^* | | 167.87 | 35.30 | 97.29 | 31.48 |  | *L_r_^*^* | 165.44 | 40.30 | 105.81 | 28.58 |
|  | *a*f*^*^* | | 143.17 | 102.38 | 129.91 | 11.43 |  | *a_r_^*^* | 143.17 | 105.54 | 129.10 | 9.53 |
|  | *b*f*^*^* | | 187.42 | 138.34 | 158.06 | 10.40 |  | *b_r_^*^* | 181.82 | 140.85 | 155.74 | 7.29 |
|  | *StdL*f*^*^* | | 54.58 | 16.85 | 31.29 | 6.08 |  | *StdL_r_^*^* | 50.99 | 14.49 | 28.88 | 5.84 |
|  | *Stda*f*^*^* | | 11.76 | 1.70 | 4.05 | 1.80 |  | *Stda_r_^*^* | 9.14 | 1.19 | 3.40 | 1.38 |
|  | *Stdb*f*^*^* | | 17.19 | 3.72 | 7.42 | 1.97 |  | *Stdb_r_^*^* | 12.96 | 2.61 | 5.79 | 1.89 |
|  | *ASM*f | | 0.93 | 0.52 | 0.77 | 0.10 |  | *ASM_r_* | 0.93 | 0.52 | 0.78 | 0.10 |
|  | *CON*f | | 0.07 | 0.01 | 0.02 | 0.01 |  | *CON_r_* | 0.06 | 0.01 | 0.02 | 0.01 |
|  | *Corr*f | | 0.96 | 0.84 | 0.90 | 0.02 |  | *Corr_r_* | 0.96 | 0.82 | 0.89 | 0.02 |
|  | *Entr*f | | 0.88 | 0.14 | 0.34 | 0.13 |  | *Entr_r_* | 0.88 | 0.10 | 0.36 | 0.14 |
|  | *IDM*f | | 1.00 | 0.95 | 0.98 | 0.01 |  | *IDM_r_* | 1.00 | 0.95 | 0.98 | 0.01 |
| Leaf features | *LP* | Lower leaves, Middle leaves, Upper leaves | | | | | | | | | | |
|  | *AP* | Wilting period, Yellowing period, Browning period, Fixation period, Dry tendon period | | | | | | | | | | |

**
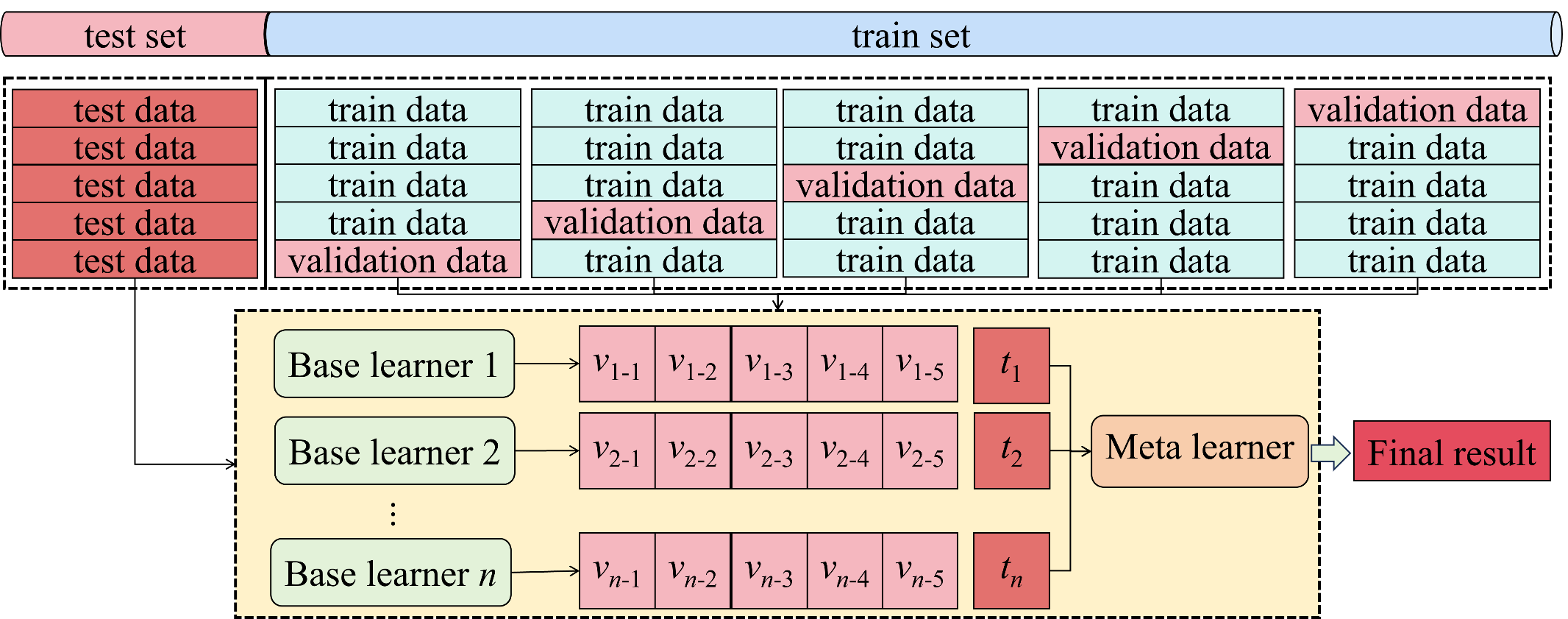
**

**Supplementary Figure 2**

Stacking ensemble learning model framework. *v_n_*_-1_-*v_n_*_-5_ represent the output result of the 5-fold cross-validation of the *n*^th^ base learner, and *t_n_* represents the prediction result of the *n*^th^ base learner on the test set.

**Supplementary Table 2.**

The hyperparameters of each machine learning model optimized by genetic algorithm

|  | Candidate machine learning models | Hyperparameters | Scope of value | Step size | Optimal hyperparameters |
| --- | --- | --- | --- | --- | --- |
| Single model | LR | \ | \ | \ | \ |
|  | SVR | C | (0.1,10) | 0.1 | 6.494 |
|  |  | gamma | (0.001,1) | 0.001 | 0.204 |
|  |  | epsilon | (0.001,1) | 0.001 | 0.012 |
|  | MLP | hidden layer size | (0,5) | 1 | 3 |
|  |  | nodes per layer | (0,20) | 1 | 10 |
|  |  | activation | [‘relu’,‘tanh’] | 1 | ‘tanh’ |
|  |  | alpha | (0.001,1) | 0.001 | 0.037 |
|  |  | batch_size | (4,128) | 4 | 24 |
| Bagging | RF | n_estimators | (50,500) | 10 | 400 |
|  |  | max_depth | (5,50) | 1 | 40 |
|  | ET | n_estimators | (50,400) | 10 | 150 |
|  |  | min_samples_split | (2,20) | 1 | 10 |
| Boosting | XGBoost | n_estimators | (50,400) | 10 | 150 |
|  |  | learning_rate | (0.001,1) | 0.001 | 0.036 |
|  |  | max_depth | (1,10) | 1 | 10 |
|  | GBDT | n_estimators | (50,400) | 10 | 170 |
|  |  | learning_rate | (0.001,1) | 0.001 | 0.044 |
|  |  | subsample | (0.001,1) | 0.001 | 0.722 |
|  | AdaBoost | n_estimators | (50,400) | 10 | 50 |
|  |  | learning_rate | (0.001,1) | 0.001 | 0.045 |
|  | LightGBM | n_estimators | (50,400) | 10 | 170 |
|  |  | learning_rate | (0.001,1) | 0.001 | 0.531 |

**Table S3.**

Five-fold cross-validation results of each candidate machine learning model

|  | *R*^2^ | | | EVS | | | MSE | | | MAE | | | MAPE | | |
| --- | --- | --- | --- | --- | --- | --- | --- | --- | --- | --- | --- | --- | --- | --- | --- |
|  | Mean | Std | CV | Mean | Std | CV | Mean | Std | CV | Mean | Std | CV | Mean | Std | CV |
| LR | 0.956 | 0.010 | 0.010 | 0.957 | 0.009 | 0.010 | 0.043 | 0.008 | 0.178 | 0.166 | 0.014 | 0.082 | 0.487 | 0.176 | 0.362 |
| MLP | 0.978 | 0.006 | 0.006 | 0.979 | 0.005 | 0.005 | 0.022 | 0.005 | 0.229 | 0.104 | 0.009 | 0.084 | 0.308 | 0.064 | 0.207 |
| SVR | 0.973 | 0.008 | 0.008 | 0.973 | 0.008 | 0.008 | 0.027 | 0.007 | 0.270 | 0.119 | 0.012 | 0.104 | 0.386 | 0.148 | 0.384 |
| RF | 0.977 | 0.005 | 0.005 | 0.978 | 0.004 | 0.004 | 0.022 | 0.004 | 0.163 | 0.101 | 0.005 | 0.051 | 0.400 | 0.128 | 0.321 |
| ET | 0.979 | 0.005 | 0.005 | 0.979 | 0.005 | 0.006 | 0.021 | 0.004 | 0.197 | 0.099 | 0.009 | 0.093 | 0.414 | 0.242 | 0.584 |
| XGBoost | 0.976 | 0.005 | 0.005 | 0.976 | 0.005 | 0.005 | 0.024 | 0.004 | 0.177 | 0.104 | 0.008 | 0.074 | 0.426 | 0.119 | 0.278 |
| AdaBoost | 0.973 | 0.006 | 0.006 | 0.974 | 0.005 | 0.005 | 0.026 | 0.005 | 0.187 | 0.116 | 0.008 | 0.071 | 0.471 | 0.188 | 0.398 |
| LightGBM | 0.975 | 0.007 | 0.007 | 0.976 | 0.007 | 0.007 | 0.025 | 0.007 | 0.270 | 0.105 | 0.008 | 0.076 | 0.294 | 0.095 | 0.322 |
| GBDT | 0.977 | 0.006 | 0.006 | 0.977 | 0.007 | 0.007 | 0.022 | 0.005 | 0.217 | 0.103 | 0.010 | 0.094 | 0.440 | 0.235 | 0.535 |





**Supplementary Figure 3.**

Prediction results of different model. **(A)** MSE. **(B)** MAE. **(C)** MAPE. **(D)** *R*^2^. **(E)** EVS.

**Supplementary Table 4**

The entropy-weighted scores of the model under different stacking strategies

|  | Model combination scheme | entropy-weighted scores | Model combination scheme | entropy-weighted scores |
| --- | --- | --- | --- | --- |
| meta learn=LR | RF+MLP+GBDT | 0.973 | RF+MLP+AdaBoost | 0.733 |
|  | ET+MLP+GBDT | 0.966 | RF+MLP+XGBoost | 0.731 |
|  | RF+MLP+LightGBM | 0.781 | ET+SVR+AdaBoost | 0.714 |
|  | ET+SVR+GBDT | 0.753 | RF+SVR+AdaBoost | 0.711 |
|  | ET+MLP+XGBoost | 0.751 | ET+SVR+LightGBM | 0.698 |
|  | RF+SVR+GBDT | 0.750 | ET+SVR+XGBoost | 0.695 |
|  | ET+MLP+LightGBM | 0.747 | RF+SVR+LightGBM | 0.686 |
|  | ET+MLP+AdaBoost | 0.739 | RF+SVR+XGBoost | 0.651 |
| meta learn=SVR | ET+MLP+GBDT | 0.903 | ET+LR+GBDT | 0.683 |
|  | RF+MLP+GBDT | 0.903 | ET+LR+AdaBoost | 0.676 |
|  | ET+MLP+AdaBoost | 0.775 | RF+LR+GBDT | 0.644 |
|  | RF+MLP+AdaBoost | 0.773 | ET+LR+LightGBM | 0.643 |
|  | RF+MLP+LightGBM | 0.727 | RF+LR+AdaBoost | 0.621 |
|  | ET+MLP+LightGBM | 0.725 | RF+LR+LightGBM | 0.609 |
|  | ET+MLP+XGBoost | 0.723 | ET+LR+XGBoost | 0.608 |
|  | RF+MLP+XGBoost | 0.707 | RF+LR+XGBoost | 0.538 |
| meta learn=MLP | RF+SVR+AdaBoost | 0.792 | RF+SVR+GBDT | 0.722 |
|  | ET+SVR+AdaBoost | 0.778 | RF+SVR+LightGBM | 0.720 |
|  | RF+LR+LightGBM | 0.771 | ET+LR+LightGBM | 0.718 |
|  | ET+LR+GBDT | 0.770 | ET+SVR+LightGBM | 0.705 |
|  | RF+LR+XGBoost | 0.765 | ET+LR+XGBoost | 0.693 |
|  | ET+LR+AdaBoost | 0.757 | ET+SVR+XGBoost | 0.682 |
|  | ET+SVR+GBDT | 0.751 | RF+MLP+XGBoost | 0.681 |
|  | RF+MLP+LightGBM | 0.731 | RF+SVR+XGBoost | 0.648 |
| Candidate base learner | MLP | 0.814 | LR | 0.540 |
|  | SVR | 0.805 | ET | 0.478 |
|  | RF | 0.765 | LightGBM | 0.464 |
|  | AdaBoost | 0.635 | XGBoost | 0.232 |
|  | GBDT | 0.558 |  |  |
